# Cell‐Based Therapy for Canavan Disease Using Human iPSC‐Derived NPCs and OPCs

**DOI:** 10.1002/advs.202002155

**Published:** 2020-10-29

**Authors:** Lizhao Feng, Jianfei Chao, E Tian, Li Li, Peng Ye, Mi Zhang, Xianwei Chen, Qi Cui, Guihua Sun, Tao Zhou, Gerardo Felix, Yue Qin, Wendong Li, Edward David Meza, Jeremy Klein, Lucy Ghoda, Weidong Hu, Yonglun Luo, Wei Dang, David Hsu, Joseph Gold, Steven A. Goldman, Reuben Matalon, Yanhong Shi

**Affiliations:** ^1^ Division of Stem Cell Biology Research Department of Developmental and Stem Cell Biology Beckman Research Institute of City of Hope 1500 E. Duarte Rd. Duarte CA 91010 USA; ^2^ Diabetes and Metabolism Research Institute at City of Hope 1500 E. Duarte Rd. Duarte CA 91010 USA; ^3^ Irell & Manella Graduate School of Biological Sciences Beckman Research Institute of City of Hope 1500 E. Duarte Rd. Duarte CA 91010 USA; ^4^ Department of Molecular Imaging and Therapy Beckman Research Institute of City of Hope 1500 E. Duarte Rd. Duarte CA 91010 USA; ^5^ Department of Biomedicine Aarhus University Aarhus 8000 Denmark; ^6^ Center for Biomedicine and Genetics Beckman Research Institute of City of Hope 1500 E. Duarte Rd. Duarte CA 91010 USA; ^7^ Center for Translational Neuromedicine University of Rochester Medical Center Rochester NY 14642 USA; ^8^ Center for Translational Neuromedicine Faculty of Health and Medical Sciences University of Copenhagen Copenhagen DK‐2200 Denmark; ^9^ Department of Pediatrics the University of Texas Medical Branch at Galveston 301 University Blvd Galveston TX 77555‐0359 USA

**Keywords:** Canavan disease, iPSCs, leukodystrophy, neural progenitor cells (NPCs), neurological diseases, oligodendrocyte progenitor cells (OPCs), stem cell therapy

## Abstract

Canavan disease (CD) is a fatal leukodystrophy caused by mutation of the aspartoacylase (*ASPA*) gene, which leads to deficiency in ASPA activity, accumulation of the substrate N‐acetyl‐*L*‐aspartate (NAA), demyelination, and spongy degeneration of the brain. There is neither a cure nor a standard treatment for this disease. In this study, human induced pluripotent stem cell (iPSC)‐based cell therapy is developed for CD. A functional *ASPA* gene is introduced into patient iPSC‐derived neural progenitor cells (iNPCs) or oligodendrocyte progenitor cells (iOPCs) via lentiviral transduction or TALEN‐mediated genetic engineering to generate ASPA iNPC or ASPA iOPC. After stereotactic transplantation into a CD (Nur7) mouse model, the engrafted cells are able to rescue major pathological features of CD, including deficient ASPA activity, elevated NAA levels, extensive vacuolation, defective myelination, and motor function deficits, in a robust and sustainable manner. Moreover, the transplanted mice exhibit much prolonged survival. These genetically engineered patient iPSC‐derived cellular products are promising cell therapies for CD. This study has the potential to bring effective cell therapies, for the first time, to Canavan disease children who have no treatment options. The approach established in this study can also benefit many other children who have deadly genetic diseases that have no cure.

## Graphical abstract

Canavan disease (CD) is a leukodystrophy caused by mutation of the aspartoacylase*(ASPA)* gene. A functional *ASPA* gene is introduced into patient induced pluripotent stem cell (iPSC)‐derived neural progenitor or oligodendrocyte progenitor cells. After transplantation into CD (Nur7) mice, the engrafted cells could rescue major pathological features of CD, including vacuolation, defective myelination, and motor function deficits, and prolonge survival.

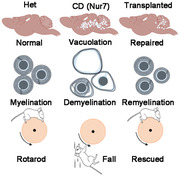

## 1 Introduction

Canavan disease (CD) is a rare, autosomal recessive neurodevelopmental disorder that affects children from infancy.^[^ ^1^ ^]^ Most children with infantile‐onset CD, the most prevalent form of the disease, will die within the first decade of life. There is neither a cure nor a standard treatment for this disease. CD is caused by genetic mutation in the aspartoacylase (*ASPA*) gene, which encodes a metabolic enzyme synthesized by oligodendrocytes in the brain.^[^ ^1^ ^]^ The ASPA enzyme breaks down N‐acetyl‐aspartate (NAA), an amino acid derivative in the brain. The cycle of production and breakdown of NAA appears to be critical for maintaining the white matter of the brain, which consists of nerve fibers covered by myelin. Mutation of the *ASPA* gene results in a deficiency in the ASPA enzyme, which in turn leads to accumulation of the NAA substrate, spongy degeneration (vacuolation), and myelination defect in the brain. The clinical symptoms of CD include impaired motor function, mental retardation, and early death.^[^ ^2^ ^]^

There is currently no approved therapy for this condition. The closest therapeutic candidate under clinical development for this disease is the delivery of a functional *ASPA* gene directly into the brain via adeno‐associated viral (AAV) transduction^[^ ^3^ ^]^ or liposome‐mediated transfection.^[^ ^4^ ^]^ The AAV product has undergone a phase 1 clinical trial with 13 patients, while the liposome *ASPA* gene transfer has been tested in 2 patients. The results of the studies showed reasonable safety profiles, however, the clinical benefits to the patients were limited.^[^ ^3^, ^4^ ^]^ There is a clear, unmet medical need for an effective therapy for CD.

Stem cell technology holds great promise for the treatment of intractable human diseases. Several clinical trials are ongoing using cells derived from human embryonic stem cells or human induced pluripotent stem cells.^[^ ^5^ ^]^ iPSCs could provide an autologous and expandable donor source for the generation of specific somatic cell types and tissues from individual patients.^[^ ^6^ ^]^ Furthermore, patient‐specific iPSCs are tailored to specific individuals, and therefore could reduce the potential for immune rejection. Neural progenitor cells (NPCs) have been used in clinical trials and shown a favorable safety profile.^[^ ^7^ ^]^ The high expandability and short differentiation time^[^ ^8^ ^]^ make iPSC‐derived NPCs (iNPCs) a desirable cell source for cell therapy.

Because CD is a demyelination disease with oligodendrocyte loss in the brain of CD patients, oligodendrocyte progenitor cells (OPCs), the precursor cells of oligodendrocytes, could also be a good candidate for CD cell therapy.^[^ ^9^ ^]^ OPCs have been successfully derived from human iPSCs.^[^ ^10^ ^]^ They are highly migratory after intracerebral engraftment, and can differentiate into oligodendrocytes and myelinate dysmyelinated loci throughout the brain.^[^ ^10^, ^11^ ^]^

In this study, we developed good manufacturing practice (GMP)‐compatible processes for human iPSC derivation, expansion, and differentiation. We generated iPSCs from CD patients and differentiated CD iPSCs into iNPCs using GMP‐compatible processes we established. A functional *ASPA* gene was introduced into CD iNPCs by lentiviral transduction. The resultant ASPA iNPCs were transplanted into the brains of an immunodeficient CD (Nur7) mouse model. The efficacy and preliminary safety of the transplanted ASPA iNPCs were evaluated. Furthermore, we introduced a wild type *ASPA* gene into a defined locus in CD iPSCs by TALEN‐mediated gene editing. These gene‐edited iPSCs were further differentiated into OPCs. The resultant ASPA iOPCs were also transplanted into CD (Nur7) mouse brains to determine their efficacy and preliminary safety.

## 2 Results

### 2.1 Manufacturing Canavan Disease Patient iPSCs and Differentiating Them into iNPCs

The objective of this study is to establish human iPSC‐based cell therapies for CD. We have demonstrated that research‐grade NPCs derived from CD patient iPSCs that were transduced with a wild type ASPA gene are able to ameliorate disease phenotypes in a CD (Nur7) mouse model in our developmental stage study. In order to move the therapeutic candidate to the clinic, we developed GMP‐compatible processes to manufacture the CD patient iPSC‐derived cellular product, in order to transfer the processes to GMP manufacturing. We established a GMP‐compatible process to derive human iPSCs by episomal reprogramming^[^ ^12^ ^]^ in an integration‐free, xeno‐free, and feeder‐free manner. We further developed methods to expand human iPSCs and differentiate them iNPCs under chemically defined, xeno‐free, and feeder‐free, GMP‐compatible conditions.

**Figure 1:**
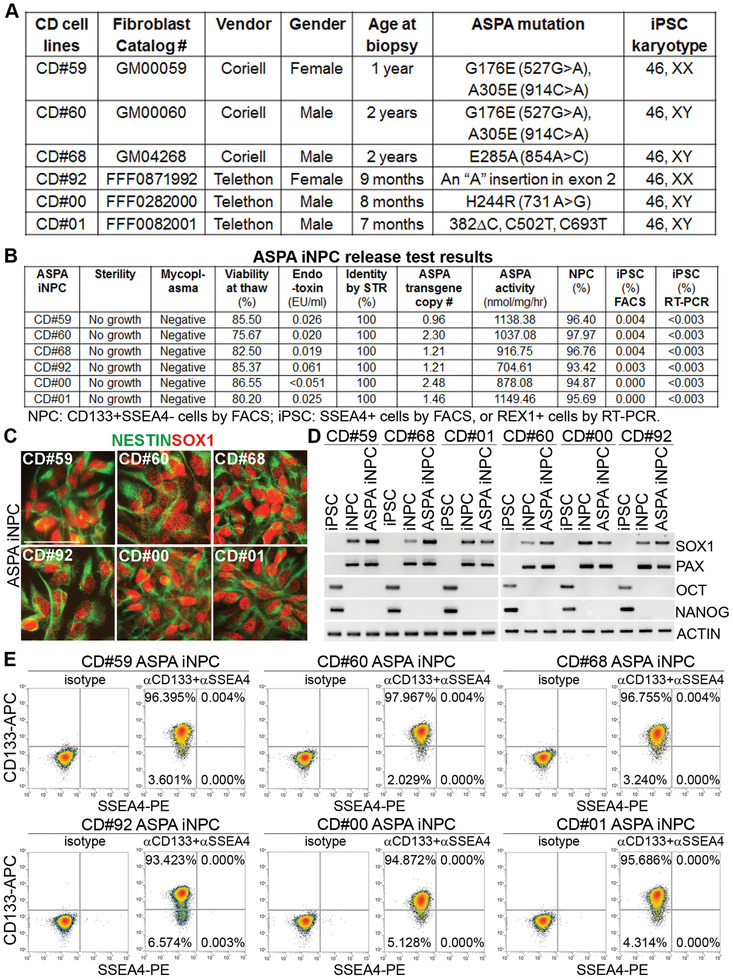
Characterization of the ASPA iNPCs. A) iPSC lines used in the study. B) The ASPA iNPC release test results. The ASPA activity was expressed as the increase of aspartic acid in nmol per mg cell lysates per hour at 37 °C. %NPC was determined as the percent of CD133^+^SSEA4^−^ cells in ASPA iNPCs by FACS. % residual iPSCs was determined as the percent of SSEA4^+^ cells by FACS or the percent of REX1^+^ cells by RT‐qPCR. C) Immunostaining of ASPA iNPCs for the NPC markers NESTIN and SOX1. Scale bar: 50 µm. D) RT‐PCR analysis of ASPA iNPCs for the expression of the NPC markers SOX1 and PAX6 and lack of expression of the pluripotency factors OCT4 and NANOG. ACTIN was included as a loading control. E) Flow cytometry analysis to determine the percentage of CD133^+^SSEA4^−^ NPC population and the residual SSEA4^+^ iPSC population in ASPA iNPCs. Isotype IgG was included as the negative control.

We derived iPSCs from fibroblasts generated from six CD patients using the GMP‐compatible manufacturing process we established. The cohort of the CD patients include patients CD#59 and CD#60 who carry the G176E and A305E mutations in the ASPA gene, patient CD#68 who carries the E285A mutation in the ASPA gene, patient CD#92 who has one nucleotide insertion in exon 2 of the ASPA gene, CD#00 who has a H244R mutation in the ASPA gene, and CD#01 who has a deletion and two point mutations in the ASPA gene (**Figure** **1**A). Among the ASPA mutations, A305E is the most common mutation (60%) in non‐Jewish CD patients,^[^ ^13^ ^]^ while E285A is the predominant mutation (accounting for over 82% of mutations) among the Ashkenazi Jewish population.^[^ ^14^ ^]^ The CD patient‐derived fibroblast cells were reprogrammed via nucleofection to generate iPSCs using episomal vectors encoding the reprogramming factors human OCT4, SOX2, KLF4, L‐MYC, and LIN28. At least three iPSC colonies with typical human embryonic stem cell (ESC) morphology and marker expression (Figure S1A, Supporting Information) were selected and expanded for each patient fibroblast line.

For each patient, one line of iPSCs that express the pluripotency genes and human ESC surface markers (Figure S1A, Supporting Information) and exhibit normal karyotype (Figure S2A, Supporting Information) was selected for in‐process testing. All six lines were negative for microbial and mycoplasma contamination (Table S1, Supporting Information). Short tandem repeat (STR) analysis confirmed that all CD iPSC clones exhibited the same STR pattern as their parental fibroblast cells on all loci tested (Table S2, Supporting Information). For each CD patient iPSC line, flow cytometry analysis showed that more than 90% cells express the pluripotency marker OCT4 and the human ESC surface marker SSEA4 (Table S3, Supporting Information).

Reverse transcription polymerase chain reaction (RT‐PCR) analysis was performed to confirm the activation of the endogenous pluripotency genes and detect any residual exogenous reprogramming factors in each CD iPSC line. The activation of the endogenous OCT4, SOX2, and NANOG gene expression was detected in iPSCs derived from each CD patient fibroblast line, whereas the exogenous reprogramming factors, OCT4, KLF4, MYC, and LIN28, were not detectable in any iPSCs by passage 6 (Figure S1B,C, Supporting Information). Sanger sequencing confirmed that each CD patient‐derived iPSC line harbored the same ASPA mutation as the corresponding CD patient (Figure S2B, Supporting Information).

After in‐process testing, the CD iPSCs that met the specifications were differentiated into CD iNPCs. The CD iNPCs lines were expanded up to passage 6. At this stage, all CD iNPC lines were tested for sterility and mycoplasma and confirmed to be free of contamination.

### 2.2 Generating ASPA iNPCs by Lentiviral Transduction of a Functional ASPA Gene into CD iNPCs

Because CD is caused by *ASPA* gene mutations, which lead to deficient ASPA enzymatic activity, a functional *ASPA* gene was introduced into CD iNPCs by transducing CD iNPCs with a lentiviral vector. The lentiviral vector consisting of the sequence of a functional human *ASPA* gene (R132G ASPA) under the control of the constitutive human EF1*α* promoter was called LV‐EF1*α*‐hASPA. The R132G mutation created outside of the catalytic center for the purpose of tracking did not disrupt the ASPA enzymatic activity, but increased ASPA activity mildly (Figure S2C, Supporting Information). The LV‐EF1*α*‐hASPA was used for genetic modification of CD iNPCs. The resultant cellular product was termed ASPA iNPCs.

The ASPA iNPCs were sampled during manufacturing (in‐process, Tables S1–S3, Supporting Information) and at final product stage (Figure 1B; and Table S4, Supporting Information) for characterization. According to the established procedures, the ASPA iNPCs were characterized for sterility, mycoplasma, viability at thaw, endotoxin, STR profiling, ASPA transgene copy#, ASPA activity, %NPC (CD133^+^SSEA4^−^ cells), and % residual iPSC (SSEA4^+^ cells by FACS and REX1^+^ cells by RT‐qPCR). The copy number of the virally transduced ASPA transgene in the ASPA iNPCs was determined by TaqMan real time PCR following a published protocol.^[^ ^15^ ^]^ The copy number of the transgene is less than five in all 6 ASPA iNPC lines. The ASPA activity was measured using a coupled enzymatic reaction^[^ ^16^ ^]^ and robust ASPA activity was detected in each ASPA iNPC line (Figure 1B).

We also characterized ASPA iNPCs to confirm that they expressed typical NPC markers PAX6, SOX1, NESTIN, and CD133. We showed that all 6 lines of ASPA iNPC lines expressed typical NPC markers, including NESTIN, SOX1, and PAX6, as revealed by immunostaining (for NESTIN and SOX1) and RT‐PCR (for SOX1 and PAX6) analyses (Figure 1C,D), whereas no expression of the pluripotency factors OCT4 and NANOG was detected in ASPA iNPCs (Figure 1D). FACS analysis was performed to determine the percentage of CD133^+^SSEA4^−^ NPC population, which ranged from 93.42% to 97.97% in six lines of ASPA iNPCs, and confirmed the lack of residual iPSCs in ASPA iNPCs (0–0.004% by SSEA4 FACS and <0.003% by REX1 RT‐qPCR) (Figure 1E). ASPA iNPCs derived from 6 CD patients all met the release testing criteria. In summary, we have successfully established GMP‐compatible manufacturing processes and generated genetically modified ASPA iNPCs from CD patients using these processes.

### 2.3 Generation of Immunodeficient CD (Nur7) Mice for ASPA iNPC Transplantation

The Aspa^nur7/nur7^ mouse contains a nonsense mutation (Q193X) in the ASPA gene.^[^ ^17^ ^]^ Because Aspa^nur7/nur7^ mice exhibit key pathological phenotypes resembling those of CD patients, including loss of ASPA enzymatic activity, elevated NAA levels, and extensive spongy degeneration in various brain regions,^[^ ^17^ ^]^ it is considered a relevant animal model for CD. Therefore, the Aspa^nur7/nur7^ mouse provides an excellent platform for testing the therapeutic effects of the ASPA iNPCs.

Because we needed to transplant human cells into CD (Nur7) mice, we generated an immunodeficient ASPA^nur7//nur7^ mouse model by breeding the Aspa^nur7/nur7^ mice with immunodeficient Rag2^−/−^ mice, which lack mature B and T lymphocytes.^[^ ^18^ ^]^ The resultant Aspa^nur7/nur7^/Rag2^−/−^ mice were termed “CD (Nur7) mice” for short. These mice exhibit a range of pathological features of CD (see results below) and were used for transplantation studies to evaluate the efficacy of the ASPA iNPC cellular product. All CD (Nur7) mice used for transplantation were verified to carry homozygous *nur7* and *Rag2* genetic mutations by genotyping. Postnatal day (PND) 1–4 pups of both sexes were used for transplantation.

### 2.4 The Distribution and Cell Fate of ASPA iNPCs in the Transplanted CD (Nur7) Mouse Brains

**Figure 2:**
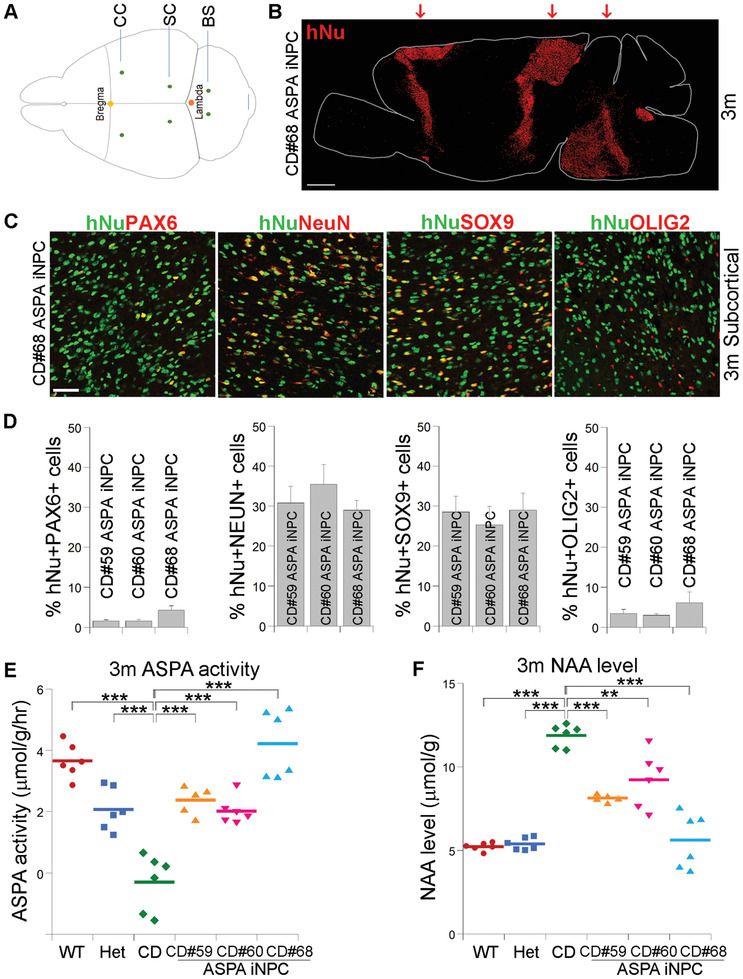
Elevated ASPA activity and reduced NAA level in ASPA iNPC‐transplanted CD (Nur7) mouse brains. A) Illustration of the injection sites in mouse brains. The bilateral injection sites were indicated in green dots. B) The ASPA iNPCs were distributed around the injection sites in the transplanted CD (Nur7) mouse brains 3 months after transplantation. The dot map of the human nuclear antigen (hNu) staining is shown. The injection sites were indicated by arrows. Scale bar: 1 mm. C) The ASPA iNPCs gave rise to neurons, astrocytes, and oligodendroglial lineage cells in the transplanted mouse brains. 3 months after transplantation, the ASPA iNPC‐transplanted brains were immunostained for hNu and the NPC marker PAX6, the neuronal marker NeuN, the astrocyte marker SOX9, and the oligodendroglial lineage marker OLIG2, respectively. The images from the subcortical white matter was shown. Scale bar: 50 µm. D) The percentage of hNu^+^ and the neural lineage marker^+^ cells in the transplanted brains. *n* = 9 fields from 3 mice for each group. E,F) Elevated ASPA activity E) and reduced NAA level F) in ASPA iNPC‐transplanted CD (Nur7) mouse brains 3 months after transplantation. The NAA level was measured using NMR. The ASPA activity was measured by NMR and expressed as reduced NAA level per gram of brain tissue within an hour (h) (µmol g^−1^ h^−1^). Each dot represents the result from an individual mouse. *n* = 6 mice for the WT, Het, and CD (Nur7) mice, 5 for the CD#59 ASPA iNPC, and 6 for the CD#60 ASPA iNPC and CD#68 ASPA iNPC‐transplanted mice, respectively. Error bars are SE of the mean. ***p < 0.001 by one‐way ANOVA followed by Dunnett's multiple comparisons test for panels (E,F).

Three lines of ASPA iNPCs derived from three different CD patients, including CD#59, CD#60, and CD#68, were injected into CD (Nur7) mouse brains individually. The injection was performed bilaterally into six sites. The injection sites include the corpus callosum, the subcortical white matter, and the brain stem (**Figure** **2**A). The ASPA iNPC‐transplanted mice were evaluated at organismal, histological, and biochemical levels. The wild type (WT, ASPA^+/+^ / Rag2^−/−^) and/or heterozygous (Het, ASPA^nur7/+^ / Rag2^−/−^) mice were included as the positive control, while the non‐transplanted CD (Nur7) mice (ASPA^nur7/nur7^ / Rag2^−/−^) were included as the negative control for the preclinical efficacy studies. In addition, the medium for ASPA iNPCs was injected into CD (Nur7) mouse brains using the same coordinates and procedure as for cell transplantation as a sham control.

First, we determined the survival, distribution, and cell fate of the ASPA iNPCs in brains of the transplanted mice by immunohistochemical staining for human nuclear antigen (hNu) and markers of various neural lineage cells. 3 months after transplantation, brains of the transplanted mice were harvested. The survival of the transplanted ASPA iNPCs was determined by immunostaining the transplanted mouse brains for hNu. We were able to detect the signal of hNu in multiple regions of the transplanted brain, including the corpus callosum, the subcortical region, and the brain stem region (Figure S3A, Supporting Information). The ASPA iNPCs were distributed around the injection sites, without extensive migration, in the transplanted CD (Nur7) mouse brain (Figure 2B).

Double staining of the transplanted brains with antibodies for hNu and the NPC marker PAX6 revealed that a small portion of the ASPA iNPCs was maintained as NPCs (Figure 2C,D; and Figure S3A,B, Supporting Information). Double staining for hNu and the neuronal marker NeuN, the astrocyte marker SOX9, and the oligodendroglial lineage marker OLIG2, respectively, revealed that the ASPA iNPCs could give rise to neurons, astrocytes, and oligodendroglial lineage cells in the transplanted brains (Figure 2C,D; and Figure S3A,B, Supporting Information). There was no obvious difference in the fate of the transplanted cells in the regions where they were located, including the corpus callosum, the subcortical, and the brain stem white matters (Figure 2C,D; and Figure S3A,B, Supporting Information), presumably because they were all white matter tracks.

### 2.5 Increased ASPA Activity and Reduced NAA Levels in ASPA iNPC‐Transplanted CD (Nur7) Mouse Brains

Because the deficiency in ASPA enzymatic activity is the underlying cause of disease phenotypes in both CD patients and animal models, we sought to determine the ASPA enzymatic activity in ASPA iNPC‐transplanted CD (Nur7) mouse brains. 3 months after transplantation, brains of the ASPA iNPC‐transplanted mouse brains were evaluated for ASPA enzymatic activity and NAA levels. Potent ASPA enzymatic activity was detected in brains of all ASPA iNPC‐transplanted mice, compared to that in control CD (Nur7) mouse brains without transplantation (Figure 2E). In contrast, the medium‐treated CD (Nur7) mice exhibited deficient ASPA activity, similar to the control CD (Nur7) mice (Figure S4A, Supporting Information). Further comparison revealed that the ASPA activity in the ASPA iNPC‐transplanted CD (Nur7) mouse brains is similar to or higher than the ASPA activity in the Het mice. Of interest, both Het human subjects and Het CD (Nur7) mice are phenotypically normal,^[^ ^17^ ^]^ although the ASPA activity in the Het mouse brains is about 50–60% of that in the WT brains (Figure 2E). It has been shown that ASPA deficiency leads to elevated NAA level in brains of both CD patients and mouse models.^[^ ^1^, ^17^, ^19^ ^]^ Consistent with elevated ASPA enzymatic activity, we detected reduced NAA level in the ASPA iNPC‐transplanted CD (Nur7) mouse brains, compared to that in control CD (Nur7) mouse brains (Figure 2F). In contrast, the NAA level remained to be elevated in medium‐treated CD (Nur7) mouse brains (Figure S4B, Supporting Information). These results together indicate that transplantation with the ASPA iNPCs was able to rescue the deficiency of ASPA enzymatic activity and reduce NAA level, both of which are major defects in CD patients and mouse models, and that the therapeutic effect was resulted from the cell products instead of the procedure by itself because medium control exhibited no effect on either ASPA activity or NAA level.

### 2.6 Rescue of Spongy Degeneration in ASPA iNPC‐Transplanted CD (Nur7) Mouse Brains

**Figure 3:**
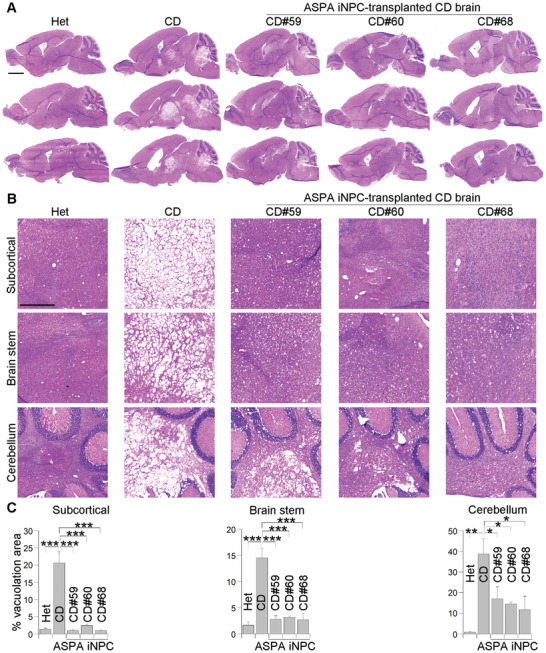
Reduced vacuolation in the ASPA iNPC‐transplanted CD (Nur7) mouse brains. A) Reduced vacuolation in brains of the ASPA iNPC‐transplanted CD (Nur7) mice 3 months after transplantation as revealed by H&E staining. Three whole brain sagittal sections of one mouse from each group are shown. The heterozygous (Het) mice were included as the positive control and the homozygous CD (Nur7) mice as the negative control. Scale bar: 2000 µm. B) Enlarged H&E images of the subcortical white matter, the brain stem and the cerebellum are shown. Scale bar: 500 µm. C) Quantification of the vacuolation area in the subcortical, the brain stem, and the cerebellum white matter. *n* = 3 mice per group. Error bars are SE of the mean. *p < 0.05, **p < 0.01, and ***p < 0.001 by one‐way ANOVA followed by Dunnett's multiple comparisons test.

Extensive spongy degeneration is a key pathological feature of CD patients and mouse models, which is revealed by vacuolation in various brain regions.^[^ ^1^, ^17^, ^19^ ^]^ Indeed, we observed extensive vacuolation in brains of the CD (Nur7) mice, compared to brains of the Het mice, which have intact brain parenchyma (**Figure** **3**A–C). In contrast, hematoxylin and eosin (H&E) staining revealed substantially reduced vacuolation in various brain regions of the ASPA iNPC‐transplanted CD (Nur7) mice, including the subcortical white matter, the brain stem and the cerebellum (Figure 3A–C), but not in medium‐treated CD (Nur7) mice (Figure S4C,D).

The extent of rescue in the cerebellum region was not as extensive as the subcortical white matter and the brain stem regions, presumably because the cerebellum is too far away from the injection sites. The ASPA iNPCs derived from three different CD patients all led to substantial rescue, in a comparable manner (Figure 3A–C). These results indicate that transplantation with ASPA iNPCs was able to rescue the spongy degeneration phenotype in CD (Nur7) mouse brains, supporting the therapeutic potential of ASPA iNPCs for their ability to ameliorate the pathological phenotypes of CD.

### 2.7 Improved Myelination in ASPA iNPC‐Transplanted CD (Nur7) Mouse Brains

**Figure 4:**
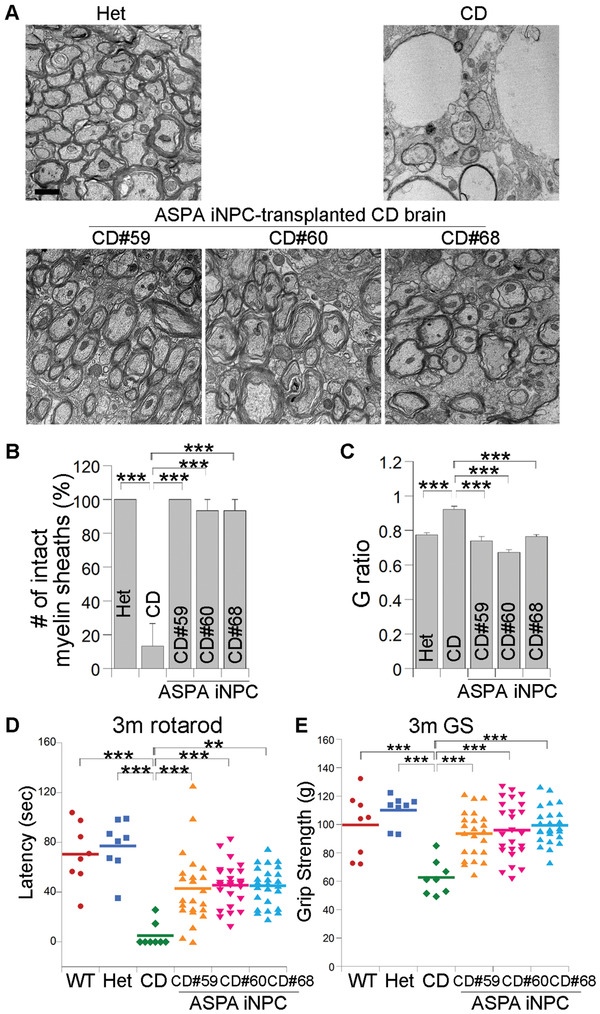
Improved myelination and motor function ASPA iNPC‐transplanted CD (Nur7) mice. A) Improved myelination in the ASPA iNPC‐transplanted CD (Nur7) mouse brains 3 months after transplantation. Improved myelination was shown by electron microscope and revealed by increased number of intact myelin sheaths and enhanced thickness of myelin sheaths in brains of the transplanted mice, compared to control CD (Nur7) mice. The subcortical white matter was processed and analyzed. Scale bar: 1 µm. B,C) Quantification showing increased number of intact myelin sheaths B) and enhanced thickness of myelin sheaths as revealed by reduced G ratio C) in brains of the ASPA iNPC‐transplanted miceCD (Nur7) mice, compared to that in control CD (Nur7) mice. *n* = 15 myelin sheaths from one mouse brain for each group. 3 transplanted brains (one brain for each line) were analyzed. Error bars are SE of the mean. D,E) Improved motor function in ASPA iNPC‐transplanted CD (Nur7) mice 3 months after transplantation revealed by rotarod D) and grip strength (GS, E) tests. Each dot represents the result from an individual mouse. *n* = 8 mice for the WT, Het, and CD (Nur7) mice, 23, 25, and 25 for the CD#59 ASPA iNPC, CD#60 ASPA iNPC, and CD#68 ASPA iNPC‐transplanted mice, respectively, for panels (D,E). *p < 0.05, **p < 0.01, and ***p < 0.001 by one‐way ANOVA followed by Tukey's multiple comparisons test for panels (B,C) and by Dunnett's multiple comparisons test.

It has been suggested that vacuolation results from myelin destruction in brains of CD (Nur7) mice.^[^ ^17^ ^]^ Consistent with the extensive vacuolation detected in brains of the CD (Nur7) mice, we observed substantially reduced number of normal myelin sheaths in brains of the CD (Nur7) mice, compared to that of the Het mice, as revealed by electron microscopy (EM) analysis (**Figure** **4**A,B) and myelin basic protein (MBP) staining (Figure S5, Supporting Information). G ratio, the ratio of the inner diameter to the outer diameter of myelin sheaths, was also altered in CD (Nur7) mouse brains. Increased G ratio was detected in brains of the CD (Nur7) mice, compared to that in the heterozygous mice (Figure 4A,C). Transplantation with ASPA iNPCs led to substantially improved myelination in CD (Nur7) mouse brains. The number of normal myelin sheaths in the ASPA iNPC‐transplanted CD brains was much higher than that in the control CD brains, reaching the level in the Het mouse brains (Figure 4A,B). Moreover, the G ratio of myelin sheaths in the transplanted brains resembled that in the Het mouse brains, both of which were much lower than that in that in the control CD brains (Figure 4A,C), indicating that the myelin sheaths in the transplanted brains are thicker than those in the untreated control CD brains. The reduced myelin sheaths and disordered nerve tracts could also be found in CD (Nur7) mouse brains by immunostaining for MBP, a marker of myelination (Figure S5, Supporting Information). Transplantation with the ASPA iNPCs improved myelination as revealed by enhanced MBP staining and better‐organized nerve tracks (Figure S5, Supporting Information).

### 2.8 Rescue of Gross Motor and Neuromuscular Function in ASPA iNPC‐Transplanted CD (Nur7) Mice

Defect in motor performance is typical of CD patients and animal models.^[^ ^1^, ^17^, ^19^ ^]^ To determine if transplantation with the ASPA iNPCs could rescue the defective motor performance in CD (Nur7) mice, we tested the ASPA iNPC‐transplanted CD (Nur7) mice in two motor skill paradigms at 3 months after transplantation. First, the transplanted mice were tested using an accelerating rotarod, a device that is designed for testing motor coordination and balance.^[^ ^20^ ^]^ Transplantation with ASPA iNPCs improved rotarod performance substantially in CD (Nur7) mice transplanted with any of the three ASPA iNPC lines, compared to the control CD (Nur7) mice (Figure 4D). A grip strength test was performed to evaluate the forepaw strength as an indication of neuromuscular function,^[^ ^21^ ^]^ using a grip strength meter. Substantial enhancement of the grip strength was also detected in CD (Nur7) mice, compared to that in the control CD (Nur7) mice transplanted with any of the three lines of ASPA iNPCs (Figure 4E). In contrast, treatment with the medium control exhibited no effect on either the rotarod performance or the grip strength of the CD (Nur7) mice (Figure S4E, F, Supporting Information). These results indicate that the ASPA iNPCs can substantially improve motor functions in a mouse model of CD. These results together provide a proof‐of‐concept that the ASPA iNPCs have great therapeutic potential to ameliorate the pathological phenotypes of CD.

### 2.9 Sustained Rescue of Disease Phenotypes in ASPA iNPC‐Transplanted CD (Nur7) Mice

The ASPA iNPCs were sustained in brains of the transplanted mice 6 months after transplantation and the cell fate was largely maintained (Figure S6A,B, Supporting Information), although there is a mild increase in the astrocyte (hNu^+^SOX9^+^) and the oligodendroglial (hNu^+^OLIG2^+^) populations, and a mild reduction in the NPC (hNu+PAX6+) and neuronal (hNu+NeuN+) populations from the transplanted cells 6 months post‐transplantation, compared to 3 months post‐transplantation (Figure S6C, Supporting Information).

**Figure 5:**
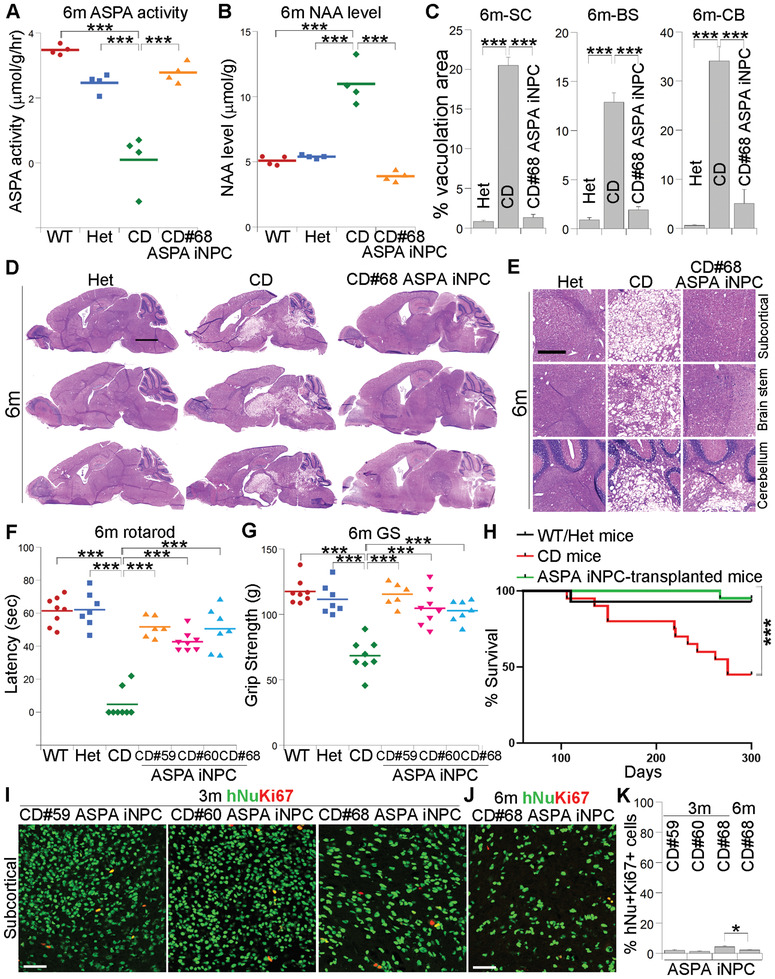
Sustained efficacy of ASPA iNPCs in transplanted CD (Nur7) mice 6 months after transplantation. A,B) Elevated ASPA activity A) and reduced NAA level B) in ASPA iNPC‐transplanted CD (Nur7) mouse brains 6 months after transplantation. The ASPA activity and NAA level was measured using NMR as described earlier. *n* = 4 mice for each group. C–E) Reduced vacuolation in brains of ASPA iNPC‐transplanted CD (Nur7) mouse brains as revealed by H&E staining. Quantification is shown in panel (C), and enlarged H&E images are shown in panel (E). *n* = 3 mice for each group. Scale bar: 2000 µm for D and 500 µm for (E). F,G) Improved motor function in ASPA iNPC‐transplanted CD (Nur7) mice 6 months after transplantation, as revealed by rotarod F) and grip strength (GS, G) tests. *n* = 8 mice for WT, Het and CD (Nur7) mice, respectively, 6 for CD#59 ASPA iNPC, 8 for CD#60 ASPA iNPC, and 7 for CD#68 ASPA iNPC‐transplanted mice. H) Life span of ASPA iNPC‐transplanted CD (Nur7) mice. The survival of the transplanted mice was monitored over 10 months. The CD (Nur7) mice were included as the negative control and the WT/Het mice as the positive control. *n* = 20 for CD, 14 for WT/Het, and 20 for the transplanted mice. I,J) Low mitotic index in ASPA iNPC‐transplanted CD (Nur7) mouse brains as revealed by hNu and Ki67 costaining 3 I) or 6 months J) after transplantation. The images from the subcortical white matter was shown Scale bar: 50 µm. K) The percentage of the hNu^+^Ki67^+^ cells out of total hNu^+^ cells in the transplanted brains. *n* = 9 fields from 3 mice for each group. Error bars are SE of the mean. ***p < 0.001 by one‐way ANOVA followed by Dunnett's multiple comparisons test for panels (A–C,F,G). ***p < 0.001 by Log‐rank test between CD (Nur7) mice and ASPA iNPC‐transplanted mice for panel (H). *p < 0.05 by one‐way ANOVA followed by Dunnett's multiple comparisons test for panel (K).

To determine if transplantation with the ASPA iNPCs could lead to sustained ASPA activity, brains of the CD68 ASPA iNPC‐transplanted CD (Nur7) mouse brains were evaluated for ASPA activity 6 months after transplantation. Substantially higher ASPA enzymatic activity was detected in brains of ASPA iNPC‐transplanted CD (Nur7) mice, compared to that in control CD (Nur7) mice (**Figure** **5**A). The ASPA activity in the ASPA iNPC‐transplanted CD (Nur7) mouse brains is similar to or even slightly higher than the ASPA activity in the Het mice 6 months after transplantation (Figure 5A). Consistent with elevated ASPA enzymatic activity, we detected dramatically reduced NAA level in brains of ASPA iNPC‐transplanted CD (Nur7) mice, compared to that in control CD (Nur7) mice (Figure 5B). These results together indicate that transplantation with ASPA iNPCs was able to rescue the deficiency of ASPA enzymatic activity and reduce NAA level in a sustainable manner.

To determine is ASPA iNPC transplantation could have long‐term beneficial effect, we examined brains of the ASPA iNPC‐transplanted CD (Nur7) mice for vacuolation. Substantially reduced vacuolation in various brain regions of the CD#68 ASPA iNPC‐transplanted CD (Nur7) mice, including the subcortical white matter, the brain stem, and the cerebellum, was detected 6 months after transplantation (Figure 5C,D,E). These results indicate that transplantation with the ASPA iNPCs was able to rescue the spongy degeneration phenotype in CD (Nur7) mouse brains in a sustainable manner.

To determine if transplantation with the ASPA iNPCs could lead to sustained improvement of motor function in CD (Nur7) mice, we tested the ASPA iNPC‐transplanted CD (Nur7) mice at 6 months after transplantation. The ASPA iNPCs improved rotarod performance in transplanted CD (Nur7) mice substantially 6 months after transplantation, compared to the control CD (Nur7) mice (Figure 5F). Considerable enhancement of the grip strength was also detected in the ASPA iNPC‐transplanted CD (Nur7) mice 6 months after transplantation, compared to the control CD (Nur7) mice (Figure 5G). This result indicates that the engrafted ASPA iNPCs can sustain improved motor functions in CD (Nur7) mice.

### 2.10 The ASPA iNPC‐Transplanted Mice Exhibit Prolonged Survival

We monitored the ASPA iNPC‐transplanted CD (Nur7) mice for up to 10 months to track their life span. The WT and Het mice were included as the positive control and the CD (Nur7) mice as the negative control. We observed substantially prolonged lifespan in the ASPA iNPC‐transplanted mice, compared to the control CD (Nur7) mice (Figure 5H). While 45% of the control CD (Nur7) mice (*n* = 20) died before 10 months, only one ASPA iNPC‐transplanted CD (Nur7) mice out of a total of 20 transplanted mice died before 10 months. Taken together, the results from the preclinical efficacy study provide a proof‐of‐concept that the ASPA iNPCs have great therapeutic potential to ameliorate the pathological phenotypes of CD in a robust and sustainable manner.

### 2.11 Preliminary Safety of the ASPA iNPCs in the Transplanted CD (Nur7) Mice

For a preliminary safety study, CD (Nur7) mice transplanted with the ASPA iNPCs were monitored monthly for up to 10 months, and no signs of tumor formation or other adverse effects were observed. At the end of 3 and 6 months, brains of the transplanted mice were harvested and analyzed. No tumor tissue was found in the transplanted brain sections. The lack of tumor formation in the ASPA iNPC‐transplanted brains was confirmed by Ki67 staining. A low mitotic index, as revealed by the low percentage (1.35–4.32%) of hNu and Ki67 double positive (hNu^+^Ki67^+^) cells out of total hNu^+^ cells, was detected in the ASPA iNPC‐transplanted brains at both 3 and 6 months post‐transplantation (Figure 5I–K; and Figure S6D, Supporting Information). Furthermore, although separate animal brains were observed at 3‐ and 6‐months after transplantation, the percent of hNu^+^Ki67^+^ cells out of total hNu^+^ cells appeared not to increase but to actually decrease in the transplanted brains, from 4.32% (at 3 months) to 2.20% (at 6 months) in CD#68 ASPA iNPC‐transplanted brains (Figure 5K). These results demonstrate preliminary safety of ASPA iNPCs in transplanted brains.

### 2.12 The ASPA iOPCs Exhibit Widespread Distribution in Transplanted CD (Nur7) Mice

**Figure 6:**
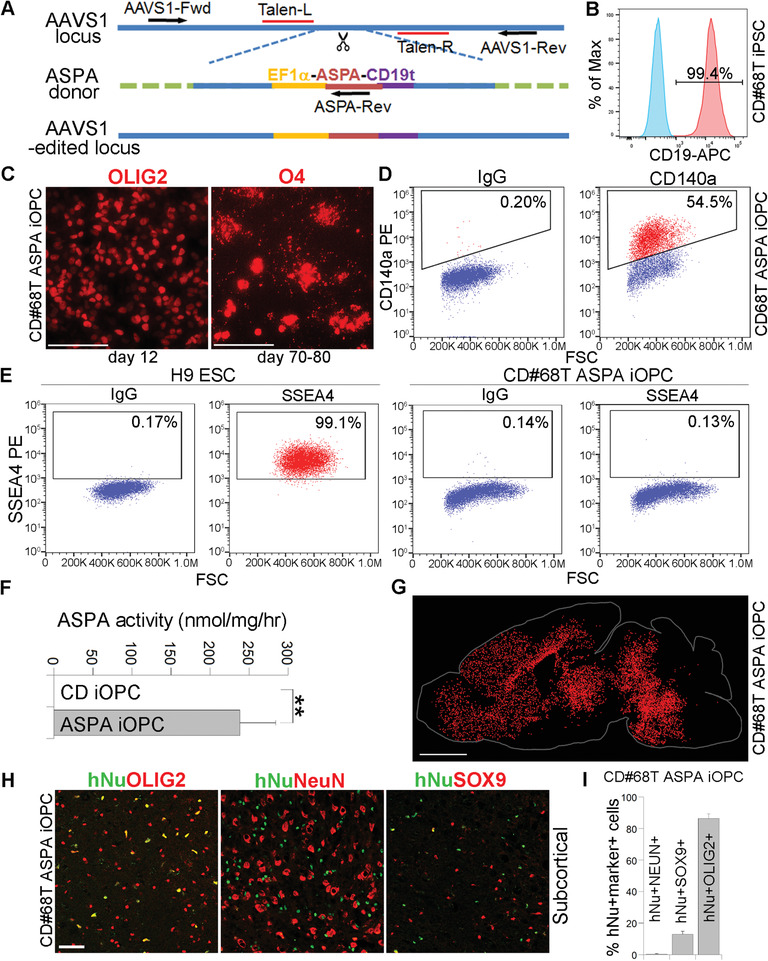
Characterization of ASPA iOPC. A) Schematic for introducing the WT *ASPA* gene into the AAVS1 locus in CD iPSCs by TALEN‐mediated gene editing. B) Flow cytometry analysis of the CD#68T‐13 ASPA iPSCs using CD19‐specific antibody. The isotype IgG was included as the negative control (blue). The ASPA‐T2A‐CD19t‐positive cells were show in red. C) Immunostaining of the CD#68T‐13 ASPA iOPCs for the oligodendroglial lineage markers OLIG2 and O4. D) Flow cytometry analysis of the ASPA iOPCs using CD140a‐specific antibody. The isotype IgG was included as the negative control. E) Lack of residual SSEA4‐positive iPSCs in ASPA iOPCs as revealed by flow cytometry. The isotype IgG was included as the negative control, which showed similar SSEA4+ population to that of SSEA4 antibody‐based flow. F) The ASPA iOPCs displayed potent ASPA enzymatic activity, compared to the control CD iOPCs. *n* = 3 replicates. **p < 0.01 by Student's *t*‐test (two tailed). G) Dot map shows widespread distribution of the transplanted ASPA iOPCs in CD (Nur7) mouse brains by immunostaining for hNu 3 months after transplantation. H) Costaining of the transplanted CD (Nur7) mouse brains for human nuclear antigen hNu and the oligodendroglial lineage marker OLIG2, the neuronal marker NeuN, or the astrocyte marker SOX9, respectively. The images from the subcortical white matter was shown. I) The percentage of the hNu^+^NeuN^+^, hNu^+^SOX9^+^, and hNu^+^OLIG2^+^ population in the ASPA iOPC‐transplanted (Nur7) mouse brains. *n* = 9 fields from 3 mice for each group. Scale bar: 100 µm for (C), 2000 µm for (G), and 50 µm for (H). Error bars are SE of the mean for panels (F,I).

As an alternative to introducing a functional *ASPA* gene into CD iNPCs through lentiviral transduction, we also knocked in a WT *ASPA* gene into the AAVS1 safe harbor site in CD68 iPSCs through TALEN‐mediated gene editing (**Figure** **6**A). The WT *ASPA* gene was linked to a truncated CD19 (CD19t) surface marker through T2A. The gene‐edited iPSCs were selected by flow cytometry using a CD19‐specific antibody. Single cell‐derived colonies were picked and expanded. One of the colonies, CD68T‐13 ASPA iPSCs, was chosen for further analysis based on colony morphology. Flow cytometry analysis using CD19‐specific antibody confirmed that the CD68T‐13 ASPA iPSC colony contained more than 99% ASPA‐CD19t‐positive cells (Figure 6B), confirming successful knock‐in. The CD68T‐13 ASPA iPSCs exhibited normal karyotype (Figure S7A, Supporting Information) and lacked off‐target mutation as revealed by whole genome sequencing (Table S5, Supporting Information).

Next we differentiated the CD68T‐13 ASPA iPSCs into iOPCs following a published protocol.^[^ ^10c,d^ ^]^ The ASPA iPSC were first differentiated into OLIG2^+^ pre‐OPCs, followed by induction into O4^+^ OPCs (Figure 6C). These ASPA iPSC‐derived OPCs were termed ASPA iOPCs. Flow cytometry analysis revealed enrichment of CD140a (PDGF*α*R)^+^ OPCs (54.5%) in the differentiated cell population^[^ ^22^ ^]^ (Figure 6D). In contrast, the CD68T ASPA iOPCs contained no detectable SSEA4^+^ residual pluripotent stem cells (0.13% detected by SSEA4 antibody minus 0.14% by IgG control) (Figure 6E). A pure population of ASPA iOPCs could be obtained by CD140a‐directed magnetic‐activated cell sorting. The ASPA iOPCs exhibited potent ASPA enzymatic activity, compared to control CD68 iOPCs without ASPA knock‐in (Figure 6F).

The ASPA iOPCs were then transplanted into brains of CD (Nur7) mice for efficacy evaluation using the same procedure as used for ASPA iNPC transplantation (Figure 2A). The distribution and cell fate of the engrafted ASPA iOPCs were analyzed 3 months after transplantation. In contrast to the ASPA iNPCs, the ASPA iOPCs showed widespread distribution throughout the brain as evidenced by immunostaining with hNu at 3 months after transplantation (Figure 6G). The ASPA iOPCs were detected in the forebrain, the subcortical, and the brain stem regions, although not the cerebellum, which may be too far away from the injection sites. Costaining for hNu and different cell lineage markers revealed that most donor cells were oligodendroglial lineage cells. The proportion of hNu^+^OLIG2^+^ cell reached 86.35 ± 2.90%. The remaining transplanted cells mostly became astrocytes (12.92 ± 1.97% hNu^+^SOX9^+^ cells). Only very few human cell‐derived neurons were detected in the transplanted brain (0.33 ± 0.33% hNu^+^NeuN^+^ cells) (Figure 6H,I; and Figure S7B,C, Supporting Information). These results indicate that the ASPA iOPCs could migrate and gave rise to oligodendroglial lineage cells in the transplanted brains.

### 2.13 The ASPA iOPCs Exhibit Robust Efficacy and Preliminary Safety in Transplanted CD (Nur7) Mice

**Figure 7:**
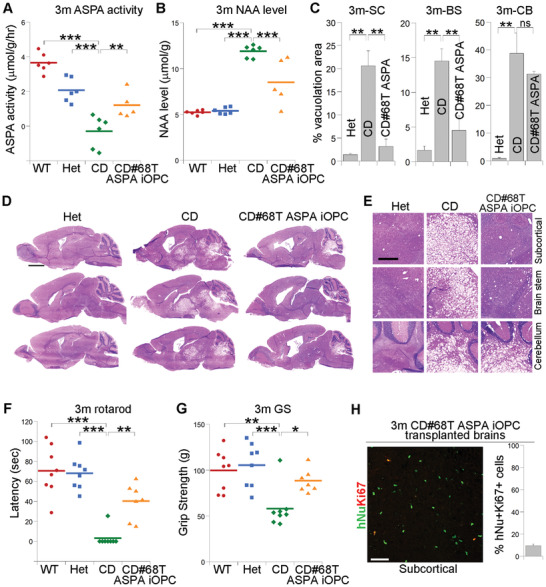
The ASPA iOPCs rescued multiple deficits in CD (Nur7) mice. A,B) Elevated ASPA activity A) and reduced NAA level B) in ASPA iOPC‐transplanted CD (Nur7) mouse brains 3 months after transplantation measured by NMR. The ASPA activity was expressed as reduced NAA level per gram of brain tissue in an hour (µmol g^−1^ h^−1^). The same data for the WT, Het, and CD (Nur7) mice from Figure 2E,F as were included here as controls. Each dot represents the result from an individual mouse for panels (A,B). *n* = 6 mice for WT, Het, and CD (Nur7) mice, respectively, and 5 for the CD#68T ASPA iOPC‐transplanted mice. C–E) Reduced vacuolation in brains of ASPA iOPC‐transplanted CD (Nur7) mouse brains as revealed by H&E staining. Quantification is shown in panel (C), and enlarged H&E images are shown in panel (E). *n* = 9 fields from 3 mice for panel (C). Scale bar: 2000 µm for (D) and 500 µm for (E). F,G) Improved motor function in ASPA iOPC‐transplanted CD (Nur7) mice 3 months after transplantation as revealed by rotarod F) or grip strength (GS, G) test. Each dot represents the result from an individual mouse for panels (F,G). *n* = 8 mice for WT, Het, and CD (Nur7) mice, respectively, and 7 for the CD#68T ASPA iOPC‐transplanted mice. The same data for the WT mice from Figure 2C were included here as a control. H) The ASPA iOPCs showed low mitotic index in transplanted mouse brains as revealed by hNu and Ki67 costaining and the low percentage of the hNu^+^Ki67^+^ cells out of total hNu+ cells. The images from the subcortical white matter was shown. *n* = 9 fields from 3 mice for panel (H). Error bars are SE of the mean. *p < 0.05, **p < 0.01, and ***p < 0.001 by one‐way ANOVA followed by Dunnett's multiple comparisons test for panels (A–C,F,G). ns stands for not statistically significant (*p* < 0.05).

To determine the efficacy of ASPA iOPCs, we transplanted ASPA iOPCs into CD (Nur7) mice and the transplanted mice were evaluated 3 months after transplantation. Biochemically, the ASPA iOPCs was able to reconstitute ASPA enzymatic activity and reduce NAA level in the transplanted CD (Nur7) mouse brains (**Figure** **7**A,B). The spongy degeneration was also rescued substantially in brains of the ASPA iOPC‐transplanted CD (Nur7) mice, compared to the control CD (Nur7) mice (Figure 7C–E). Transplantation with the ASPA iOPCs also improved myelination in CD (Nur7) mice brains as revealed by enhanced MBP staining (Figure S5, Supporting Information). Moreover, the motor function in the ASPA iOPC‐transplanted CD (Nur7) mice was improved considerably, as revealed by increased latency on the rotarod (Figure 7F) and enhanced grip strength (Figure 7G), compared to the control CD (Nur7) mice. These results indicate that ASPA iOPCs have the potential to ameliorate the pathological phenotypes of CD.

Importantly, no sign of tumor formation or other adverse effect was observed during 3 months after ASPA iOPC transplantation. Ki67 staining showed minimal number of hNu^+^Ki67^+^ cells out of total hNu^+^ cells in the ASPA iOPC‐transplanted brains (Figure 7H; and Figure S7D, Supporting Information). These results together demonstrate robust preclinical efficacy and preliminary safety of the ASPA iOPC cell product for CD therapy development.

## 3 Discussion

CD is a devastating neurological disease that has neither a cure nor a standard treatment.^[^ ^23^ ^]^ In this study, we established human iPSC‐based cell therapeutic candidates for CD. To facilitate the transfer of the cell therapeutic candidates to the clinic, we first established GMP‐compatible processes for human iPSC derivation, expansion, and differentiation. We then generated iPSCs from CD patient fibroblast cells and differentiated these iPSCs into iNPCs using the GMP‐compatible processes we established. To reconstitute ASPA activity which is deficient in both CD patients and mouse models, we developed ASPA iNPCs by introducing a functional *ASPA* gene through lentiviral transduction. We transplanted the ASPA iNPCs into CD (Nur7) mouse brains and showed that these cells were able to improve the disease symptoms dramatically, as revealed by increased ASPA activity, decreased NAA levels, substantially reduced spongy degeneration in various brain regions, and rescued motor functions of the transplanted mice. The therapeutic effect is long‐lasting, showing no diminishing effect by 6 months compared to 3 months post‐transplantation. Moreover, the transplanted CD (Nur7) mice exhibited much prolonged survival.

As an alternative strategy to introducing a functional *ASPA* gene by lentiviral transduction at the iNPC stage, we introduced a WT *ASPA* gene together with a truncated CD19 (CD19t) into the AAVS1 safe harbor site in CD iPSCs through TALEN‐mediated gene editing. The CD19t sequence has been used in a previous clinical trial and confirmed to be safe.^[^ ^24^ ^]^ The CD19t tag provides a cell surface marker for in vivo tracking of transplanted cells in patient brains by flow cytometry and immunohistochemistry approaches and can induce cell elimination through antibody‐dependent cellular cytotoxicity in case of adverse tumorigenic events.^[^ ^24^, ^25^ ^]^ We chose TALEN‐based editing for introducing the WT *ASPA* gene into CD iPSCs to generate the ASPA iOPC cell product because of the low off‐target activity associated with TALEN.^[^ ^26^ ^]^ Indeed, our whole genome sequencing revealed no off‐target effects in the top 99 potential off‐target sites. The TALEN‐edited ASPA iPSCs were differentiated into iOPCs using an established protocol.^[^ ^10^, ^11^ ^]^ After being transplanted into CD (Nur7) mouse brains, these cells showed an ability to rescue the CD phenotype that is comparable to that of ASPA iNPCs. Moreover, the ASPA iOPCs had better migration and more than 80% transplanted ASPA iOPCs went to the oligodendroglial lineage. Importantly, no tumorigenesis or other adverse effect was observed in mice transplanted with either the ASPA iNPCs or the ASPA iOPCs. These results indicate that the ASPA iNPCs and the ASPA iOPCs both have the potential to serve as cell therapy candidates for CD.

Great efforts have been directed toward therapeutic development for CD. While most other approaches resulted in limited functional recovery, gene therapy seems a promising clinical option for CD.^[^ ^23b^ ^]^ When the WT human *ASPA* gene was delivered into brains of CD animal models by recombinant adeno‐associated virus (rAAV), encouraging results were seen.^[^ ^3^, ^4^, ^27^ ^]^ However, the early clinical trial using AAV to deliver the *ASPA* gene into CD patient brains was unable to reach the desired therapeutic efficacy, although the safety profile was good.^[^ ^3^ ^]^ Recent studies showed that knockdown of the neuronal NAA‐synthesizing enzyme Nat8l by antisense oligonucleotide or AAV‐delivered shRNA to reduce NAA level improved disease phenotypes in ASPA^nur7/nur7^ mice,^[^ ^28^ ^]^ suggesting that targeting Nat8l could be a candidate approach to treat CD, although how to achieve sustained efficacy using this approach remains to be addressed.

Compared to direct gene therapy, the combined cell and gene therapy approach used in this study allowed extensive in vitro characterization of the genetically modified cells before applying these cells to in vivo study. The ASPA iNPCs were examined for transgene copy number and all six ASPA iNPC lines had less than five copies of the transgene. The ASPA iPSCs that underwent TALEN‐mediated gene editing were subjected to whole genome sequencing to make sure there were no adverse off‐target effects before differentiation and transplantation. Furthermore, the lentivirus or TALEN‐introduced *ASPA* transgene will likely be more stable because of integration events, therefore allowing sustained ASPA activity in the host brains, unlike AAV‐mediated transgene delivery which is episomal, thus can have more transient expression. Our patient iPSC‐derived autologous cellular products could also avoid potential immunogenicity associated with the AAV vector,^[^ ^29^ ^]^ and have the added benefit of regenerative potential linked to cell therapy.^[^ ^5b^ ^]^

NPCs have been used in clinical trials and shown a favorable safety profile.^[^ ^7a–d^ ^]^ NPCs isolated from human fetal brains have been transplanted into Pelizaeus–Merabacher disease patient brains and exhibited long‐term safety after 5 years of follow up.^[^ ^7^, ^30^ ^]^ No tumors or other long‐term adverse effects were observed.^[^ ^7c^ ^]^ Besides the favorable safety profile, the expandability and short manufacturing protocol make iNPCs a relatively economic and accessible cell source for cell therapy.

OPCs are another desirable cell therapy candidate for leukodystrophies including Canavan disease.^[^ ^9^, ^31^ ^]^ This study and previous studies^[^ ^10^, ^32^ ^]^ have shown that OPCs can migrate widely after intracerebral transplantation, rendering OPCs a desired vector for widespread delivery. Moreover, it has been shown that the transplanted OPCs can differentiate into oligodendrocytes and myelinate dysmyelinated loci throughout the brain.^[^ ^10^, ^11^, ^32^ ^]^ In this study, we showed that the ASPA iOPCs can migrate out of the injection sites, and rescue disease phenotypes dramatically in a leukodystrophy mouse model. However, compared to iNPCs, the differentiation protocol for iOPCs is more complex (requiring multiple growth factors), more time‐consuming and costly. It takes about 70 days or more to differentiate from human iPSCs to iOPCs,^[^ ^10a,c^ ^]^ whereas differentiation from human iPSCs to iNPC only needs 8 days.^[^ ^8^ ^]^ Moreover, the iNPCs are of high purity and can be easily expanded to produce enough cells for human applications.^[^ ^30^ ^]^ The current protocol for iOPC differentiation can only produce limited number of cells and iOPCs are not as easy to maintain and expand. Further optimized protocol for iOPC differentiation with shorter differentiation time, simpler procedure with less expensive reagents, and higher differentiation efficiency may facilitate the application of iOPCs into the clinic in the future.

It is worth noting that although the ASPA iNPCs did not migrate in the brain after transplantation, they were able to rescue the disease phenotypes in a robust and sustainable manner. One explanation for these unexpected results is because NAA travels in the brain through an intercompartmental cycling via extracellular fluids, between its anabolic compartment in neurons and catabolic compartment in oligodendrocytes^[^ ^33^ ^]^ or transplanted ASPA iNPCs in this case. After NAA is released from neurons, it can move to the transplanted cells that have ASPA activity through a concentration gradient, therefore leading to widespread reduction of NAA level, and consequently extensive rescue of spongy degeneration and myelination defect in the brain.

Unlimited source of cells derived from iPSCs and the low risk of immune rejection associated with autologous cell transplantation render human iPSC‐based autologous cellular products great potential for regenerative medicine.^[^ ^5b^ ^]^ Indeed, the first clinical study using human iPSC‐based product was initiated in 2014, in which autologous retinal pigment epithelium sheets derived from patient's own iPSCs were transplanted back to the patient. This treatment has resulted in favorable outcome, halting macular degeneration in the absence of anti‐VEGF drug administration.^[^ ^34^ ^]^

Despite the huge advantage associated with human iPSC‐derived cellular products, there remain issues related to iPSC‐based cell therapy, including teratoma formation and high cost of individualized cell products. To address the safety concern associated with potential development of teratoma from iPSC products, we developed an standard operation procedure (SOP) that allows efficient and reproducible differentiation of iPSCs into iNPCs with undetectable residual iPSCs. Whether there were any residual iPSCs in ASPA iNPCs was tested using both FACS analysis and RT‐qPCR assay and a stringent release specification was set for the ASPA iNPC products. The residual iPSCs in all six ASPA iNPC products were below the detection limit for both FACS and RT‐qPCR analyses. Furthermore, continuous monitoring of the ASPA iNPC‐transplanted mice for up to 10 months and the ASPA iOPC‐transplanted mice for 3 months revealed no sign of tumorigenesis. These results suggest the preclinical safety of our cellular products.

The use of autologous iPSCs as the source of cell therapy products comes at high cost. Ideally, an off‐the‐shelf allogenic product would address this concern. The use of allogeneic iPSCs, in which a single lot of cells could be used to treat multiple patients, would bring down the cost for iPSC‐based cell product manufacturing. However, this would come at the price of immune rejection caused by human leukocyte antigen (HLA) mismatching and, thus, poses a major challenge for allogeneic transplantation. The rejection issue has typically been addressed through immunosuppression, which has been effective but can itself be costly and its serious side effects for long term application^[^ ^35^ ^]^ would further complicate the management of these CD patients. The approach taken in Japan by using iPSC stocks from HLA homozygous donors to cover most HLA haplotypes^[^ ^36^ ^]^ would not likely be effective in CD which is associated with a diverse genetic background. An alternative approach manipulates the immune responses through gene editing to overcome immune rejection associated with allogeneic transplantation.^[^ ^37^ ^]^ This approach has great potential to generate universal donor cells, but brings its own safety concerns, for example, the potential of increased tumorigenicity due to compromised immune surveillance. From the immunological point of view, autologous transplantation is ideal for cell therapy because these cells may avoid any potential immune‐mediated complications. It is anticipated that the cost of iPSC‐based cell therapy manufacturing will be reduced with the availability of low‐cost reagents,^[^ ^38^ ^]^ and derisking of GMP manufacturing through the development of GMP‐compatible processes as described in this study that are cost‐effective and easily transferrable to GMP.

## 4 Conclusion

Leukodystrophies are among the most debilitating neurological disorders, and are appealing targets for cell therapies because there is no need for the grafted cells to integrate into the existing neural network.^[^ ^9^ ^]^ This study provides important preclinical efficacy data for developing a therapeutic candidate for CD, a devastating leukodystrophy that has neither a cure nor a standard treatment. This is the first application of human iPSC technology in developing a stem cell therapy for CD. It provides a robust proof‐of‐principle for cell therapy development of this and other diseases of this kind. The feasibility and efficacy study presented here represents a critical step toward bringing the human iPSC‐derived cellular products into the clinic for the treatment of CD and related diseases.

## 5 Experimental Section

##### CD iPSC Production

CD iPSCs were manufactured using an integration‐free, xeno‐free, and feeder‐free method by following the specific SOP was established in this study. Specifically, CD patient fibroblasts CD59 (Coriell, GM00059), CD60 (Coriell, GM00060), CD68 (Coriell, GM04268), CD92 (ID 21 282, Biobank code FFF0871992, Telethon), CD00 (ID 22 217, Biobank code FFF0282000, Telethon), and CD01 (ID 22 276, Biobank code FFF0082001, Telethon) were reprogrammed using episomal vectors expressing human OCT4, SOX2, KLF4, L‐MYC, LIN28, and p53 shRNA (sh‐p53) (Addgene plasmids pCXLE‐hSK, pCXLEhUL, pCXLE‐hOCT3/4‐shp53‐F, and pCXWB‐EBNA1, Table S6, Supporting Information) as described.^[^ ^12^ ^]^ Cells electroporated with the reprogramming vectors using 4D Nucleofector (Lonza) were seeded onto plates coated with recombinant human Laminin‐521 matrix (Thermo Fisher, A29249) and maintained in Essential 8 (E8) medium (Thermo Fisher, A1517001), a xeno‐free medium. iPSC clones were picked around day 20 and expanded in E8 medium. For immunostaining, iPSCs were passage and seeded on 12‐well Laminin‐521‐coated plates for 2–3 days. The resultant iPSC clones were ready for staining.

##### Differentiation of CD iPSCs into CD iNPCs

CD iPSCs were differentiated iNPCs on recombinant human Laminin‐521‐coated plates by following the SOP that was developed following an established protocol.^[^ ^8^ ^]^ To start neural induction, human iPSCs were dissociated into single cells, seeded onto Laminin‐521‐coated plate, and cultured in E8 medium. After 2 days, cells were switched to Neural Induction Medium 1 (NIM‐1) containing Dulbecco's Modified Eagle Medium: Nutrient Mixture F‐12 (DMEM/F12, Thermo Fisher, 11 330 032), 1x N2 (Thermo Fisher, 17 502 048), 1x B27 (Thermo Fisher, 12 587 010), 1x NEAA (Gibico, 11 140 076), 2 × 10^−3^ m GlutaMAX (Thermo Fisher, 35 050 061), 0.1 × 10^−6^ m RA (Sigma, R2625), 4 × 10^−6^ m CHIR99021 (Cellagen Technology, C2447), 3 × 10^−6^ m SB431542 (Peprocell, 04‐0010‐10), 2 × 10^−6^ m Dorsomorphin (Sigma, P5499), and 10 ng mL^−1^ hLIF (Millipore Sigma, GF342). Cells were cultured in NIM‐1 for 2 days, then switched to Neural Induction Medium 2 (NIM‐2) containing DMEM/F12, 1x N2, 1x B27, 1x NEAA, 2 × 10^−3^ m GlutaMAX, 0.1 × 10^−6^ m RA, 4 × 10^−6^ m CHIR99021, 3 × 10^−6^ m SB431542, and 10 ng mL^−1^ hLIF with daily medium change for 5 days. The resultant iNPCs were dissociated and cultured in Neural Progenitor Maintenance Medium containing DMEM/F12, 1x N2, 1x B27, 2 × 10^−3^ m GlutaMAX, 0.1 × 10^−6^ m RA, 3 × 10^−6^ m CHIR99021, 2 × 10^−6^ m SB431542, 10 ng mL^−1^ EGF (PeproTech, 100–18b), and 10 ng mL^−1^ FGF (PeproTech, 100–15), with medium change every other day. The CD iNPCs were expanded and cells before passage 6 were used. For immunostaining, dissociated single cells were seeded on Matirgel (Corning, 354 230)‐coated coverslip in 24 well plates for 2–3 days.

##### ASPA Viral Preparation and Transduction

The cloned DNA that was used for genetic modification of CD iNPCs consists of the sequence of a functional human *ASPA* gene under the control of the constitutive human EF1*α* promoter. The human ASPA coding sequence was PCR‐amplified using the ASPA cDNA clone MGC:34 517 (IMAGE: 5 180 104) as the template. The ASPA cDNA was cloned into the pSIN lentiviral vector downstream of the EF1*α* promoter. The EF1*α* promoter and the ASPA cDNA fragments were subsequently PCR‐amplified using the pSIN‐ASPA as the template and subcloned into the self‐inactivating pHIV7 lentiviral vector described previously.^[^ ^24^, ^39^ ^]^ The resultant lentiviral vector was called LV‐EF1*α*‐hASPA. In order to track the transplanted cells in patient brains, a point mutation was created in the ASPA gene by changing the codon of Arginine (AGG) at amino acid residue 132 to that of Glycine (GGG). Arginine 132 was selected for mutation because it is located outside of the catalytic center of the ASPA protein. To package the ASPA‐expressing lentivirus, the LV‐EF1*α*‐hASPA transgene vector, together with the VSV‐G, REV, and MDL packaging vectors were transfected into HEK 293T cells using the calcium phosphate transfection method as was described previously.^[^ ^40^ ^]^ 48 h after transfection, virus was harvested, concentrated by ultracentrifugation and stocked in −80 °C. For lentiviral transduction, 1.5 × 10^6^ dissociated single NPCs were seed in T25 flask and viruses were added when cells were attached. Then ASPA iNPCs were lifted and expanded in suspension culture. The ASPA iNPC before passage 6 were used for characterization and transplantation.

##### Generation of the ASPA‐CD68 iPSCs Using TALEN Editing

The ASPA‐CD68 iPSCs were generated by TALEN‐mediated gene editing. The hAAVS1 TALEN left and right vectors were used for TALEN‐mediated targeting of the AAVS1 locus as described.^[^ ^41^ ^]^ The donor vector was constructed using the AAVS1‐CAG‐hrGFP vector by inserting the EF1*α*‐ASPA‐T2A‐CD19t fragment between the AAVS1 left and right arm. The hAAVS1 TALEN left and right vectors and the donor plasmid were delivered via nucleofection into CD68 iPSCs. The transfected iPSCs were sorted by using the CD19 antibody and seeded as single cells. The single cell‐derived clones were picked and screened by PCR. Three primers: AAVS1‐ Fwd, AAVS1‐ Rev, and ASPA‐Rev, were designed for genotyping of the iPSC clones. Three iPSC clones with homozygous insertion were chosen, expanded, and stocked. The CD68T‐13 iPSC clone was randomly selected from these three colnes for further experiments. The hAAVS1 TALEN Right, hAAVS1 TALEN Left, and AAVS1‐CAG‐hrGFP vectors were gifts from Dr. Su‐Chun Zhang (Table S6, Supporting Information).

##### Whole Genome Sequencing and TALEN Off‐Target Analysis

Genomic DNA from control CD iPSCs and TALEN‐edited ASPA iPSCs were subjected to whole genome sequencing using the BGIseq 500 (MGI Tech). High quality genomic DNA was purified from the cells using Wizard SV Genomic DNA Purification System (Promega, A2360) and quantified using Qubit 3.0 fluorometer. For sequencing library generation, the genomic DNA was fragmentated into sizes of 50–800 bp using ultrasound‐based fragmentation (Covaris E220). The fragmented DNA were further selected with AMPure XP beads (Beckman Coulter, A63881) to enrich DNA of 100–300 bp, which were then repaired with a blunt ending enzyme and by addition of 3’ A overhang. A *T* tailed adapter was ligated to both ends of the DNA fragments and amplified by PCR (8 cycles). The PCR product was then denatured and annealed with a single strand bridging DNA that is reverse‐complemented to both ends of the PCR product to generate single‐strand circular DNA. The single‐strand molecule was ligated using a DNA ligase. The excessive linear molecule was digested with the exonuclease. DNA nanoballs (DNB) were then generated from the single‐strand circular DNA according to the manufacturer's instruction (MGI Tech) and sequenced with BGISEQ‐500 using pair‐end 100 cycles. For each sample, coverage of over 30X was generated. The sequences of DNBs were base called using the base calling software Zebra call. Calling for variants were carried out with BWA^[^ ^42^ ^]^ and GATK.^[^ ^43^ ^]^ Structure variation was analyzed using breakDancer (http://www.nature.com/nmeth/journal/v6/n9/abs/nmeth.1363.html). The potential off‐target sites of TALEN were predicted using a genome wide TALEN off‐target site prediction tool TALENoffer.^[^ ^44^ ^]^ A total of 100 sites including the target site and the tope 99 potential off‐target sites were export from TALENoffer. The potential off‐target sites were evaluated using whole genome sequencing. No mutation was found on any of these sites (Table S5, Supporting Information).

##### Differentiation of ASPA‐CD68 iPSCs into iOPCs

The ASPA‐CD68 iPSCs were differentiated into iOPCs by following a previously published protocol.^[^ ^10c,d^ ^]^ Briefly, ASPA‐CD68 iPSCs were dissociated into single cells and induced by OPC‐I Medium containing DMEM/F12, 1x N2, 2 × 10^−3^ m GlutaMAX, 0.1 × 10^−3^ m RA (Sigma, R2625), 10 × 10^−3^ m SB431542 (Peprocell, 04‐0010‐10), and 250 × 10^−9^ m LDN‐193189 (Peprocell, 04‐0074‐10) for 8 days. Then cells were switched to OPC‐II Medium containing DMEM/F12, 1x N2, 2 × 10^−3^ m GlutaMAX, 0.1 × 10^−6^ m RA, and 1 × 10^−6^ m SAG (Sigma, ML1314) for another 4 days. After 12 days of culture, cells were dissociated and cultured in flasks for overnight to form spheres. The resultant pre‐OPC spheres were switched to OPC‐III Medium containing DMEM/F12, 1x N2, 1x B27 minus vitamin A (Thermo Fisher, 12 587 010), 2 × 10^−3^ m GlutaMAX, 0.1 × 10^−6^ m RA, and 1 × 10^−6^ m SAGfor 8 days, and then switched to PDGF medium containing DMEM/F12, 1x N2, 1x B27 minus vitamin A, 2 × 10^−3^ m GlutaMAX, 10 ng mL^−1^ PDGF‐AA (R&D, 221‐AA‐050), 10 ng mL^−1^ IGF‐1 (R&D, 291‐GG‐01M), 5 ng mL^−1^ HGF (R&D, 294‐HG‐250), 10 ng mL^−1^ NT3 (EMD Millipore, GF031; and PeproTech, AF‐450‐03), 60 ng mL^−1^ T3 (Sigma, T2877), 100 ng mL^−1^ Biotin (Sigma, 4639), 1 × 10^−6^ m cAMP (Sigma, D0627), and 25 µg m L^−1^ Insulin (Sigma, I9278) for 10 days. After 18 days of suspension culture, the spheres were attached on Matrigel‐coated plates and cultured for 30–60 days in the PDGF medium. OPCs could be detected by flow cytometry with a CD140a antibody and by live staining with an O4 antibody after 30 days of attached culture. After 30–60 days of attached culture, OPCs were collected for transplantation.

##### Flow Cytometry

Human H9 ESCs (WiCell, WA09) were used as the positive control for FACS analysis to detect the pluripotency marker OCT4 and the human ESC cell surface marker SSEA4. HEK293T cells were used as the negative cell control for iPSC and NPC marker detection. Cells were dissociated and passed through a 70 µm cell strainer to make single cell suspension. For cell surface marker staining, cells were directly incubated with fluorophore‐conjugated primary antibodies for 20 min on ice. The same fluorophore‐conjugated IgGs were included as the isotype controls. For intracellular OCT4 staining, cells were first fixed and permeabilized using a Fixation/Permeabilization Solution Kit (BD, 554 714) before incubation with the PE‐conjugated anti‐Oct3/4 primary antibody. The PE‐conjugated mouse IgG1 was included as the isotype control. Cells were washed twice and resuspended in phosphate‐buffered saline (PBS) containing DAPI and 0.1% donkey serum. The samples were run on Attune NxT Flow Cytometer (ThermoFisher Scientific) and data were analyzed by FlowJo v10. The detailed information of all the primary antibodies and isotype controls used were listed in Table S7 (Supporting Information).

##### Immunocytochemistry

Cells were fixed with 4% paraformaldehyde (PFA) at room temperature (RT) for 10 min. After fixation, cells were washed with PBS twice and blocked with 5% donkey serum diluted in PBS with 0.1% triton (PBST) for 1 h at RT. The fixed cells were then incubated with primary antibodies at 4 °C for overnight. On the following day, cells were washed with PBS twice, incubated with secondary antibodies at RT for 1 h, and washed. Cells were counterstained with DAPI before mounting for imaging. Images were taken using Nikon ECLIPSE TE2000‐S or Nikon Ti‐2. The detailed information of the primary antibodies used was listed in Table S7 (Supporting Information).

##### Viability Assay

The vials with frozen cells were thawed in a 37 °C water bath and the content was transferred to a 15 mL conical tube. 3 mL medium was added drop by drop and the cell suspension was centrifuged at 200 × *g* for 3 min. The cell pellet was resuspended in Perfusion Fluid CNS (CMAP000151, Harvard Apparatus). A small aliquot of cell suspension was further diluted by Trypan blue solution. Live and dead cells were counted by Hemocytometer. Three cryopreserved vials were tested for each cell lines.

##### Sterility and Endotoxin Test

1–2 mL media were collected from culturing plates or flasks and sent to Department of Pathology in City of Hope to test for sterility. 1 mL media were collected from culturing plates or flasks and sent to Center for Biomedicine and Genetics and Analytical Pharmacology Core Facility of City of Hope to test for endotoxin.

##### Karyotype and STR Analysis

iPSCs in culture were directly sent to the Cyotogenetics Core of City of Hope for karyotype analysis using standard G‐banding method. Total 20 metaphase cells were analyzed for each sample. For STR assay, DNA was first purified from fibroblasts, iPSCs, and ASPA iNPCs. Geneprint 10 System PCR Amplification Kit (Promega, B9510) was used to generate a 10‐locus DNA profile that is unique to each individual. PCR products were sent to City of Hope Integrative Genomics Core for fragment analysis. The results were analyzed using the GeneMapper Software 5 (Thermo Fisher).

##### Exon Sequencing of the ASPA Genomic DNA

Genomic DNAs were extracted from CD iPSCs using QuickExtract DNA Extraction Solution (Lucigen, QE09050). The primers used for sequencing each exon were listed in Table S8 (Supporting Information).

##### ASPA Enzymatic Activity Assay for ASPA iNPCs

The ASPA enzymatic assay was developed in the laboratory based on a published protocol.^[^ ^16^, ^45^ ^]^ Cell lysates were prepared using radioimmunoprecipitation assay buffer (RIPA) buffer with phenylmethylsulfonyl fluoride (PMSF) and protein concentration was determined by Bradford. For the first reaction, 100 µg protein lysates in 50 µL RIPA buffer was mixed with 50 µL 2 x Assay Buffer I with the final concentration of 50 × 10^−3^ m Tris‐HCl, pH8.0, 50 × 10^−3^ m NaCl, 0.1 × 10^−3^ m DTT, 0.05% IGEPAL CA‐630, 2.5 × 10^−3^ m CaCl2, and 5 × 10^−3^ m NAA. The reaction mixture was incubated at 37 °C for 1 h, and the reaction was stopped by heating the tubes at 100 °C for 3 min. After centrifugation at 15 000 g for 5 min, the supernatant was collected for the second reaction. For the second reaction, 90 µL of the first reaction supernatant was added to 90 µL 2 x Assay Buffer II with the final concentration of 50 × 10^−3^ m Tris‐HCl pH 8.0, 50 × 10^−3^ m NaCl, 2.5 × 10^−3^ m alpha‐ketoglutarate (AKG), 1 mg mL^−1^ BSA, 5 × 10^−6^ m PLP, 0.5 × 10^−3^ m *β* ‐NADH, 10 units MDH, and 10 unit glutamate‐oxalacetate transaminase (GOT). 20 min later, OD340 nm was determined by luminescence reader. The ASPA activity is defined by the production of aspartate in nmol by 1 mg protein lysate in 1 h at 37 °C.

##### ASPA Transgene Copy Number Analysis

Because the human ASPA transgene in the lentiviral vector was integrated into the genome together with the PBS/psi region, the copy number of the human ASPA transgene was measured by detecting the PBS/psi region.^[^ ^46^ ^]^ Specifically, the ASPA transgene copy number was detected by TaqMan real time PCR using Step One Plus real‐time PCR system (Applied Biosystems) with primers in the PBS/psi region: PBS/psi‐Fwd and PBS/psi‐Fwd, and the PBS/psi‐TaqMan probe. The Albumin gene is a single copy gene in the genome (2 copies per cell). It was included as an internal control and amplified using primers: Albumin‐Fwd and Albumin‐Rev, and the Albumin‐TaqMan probe. The gBlock DNA fragment mixtures of psi and albumin with different ratio were amplified to create a standard curve to determine the relationship between ∆Ct (psi‐albumin) and log2(psi copy number). If the log2 (psi copy number) is n for the unknown sample, the transduced hASPA copy number/Cell = power (2, n). The Ct values were determined by TaqMan real time PCR, and used to calculate the copy numbers of both Albumin and the ASPA transgene based on the standard curves. The primers and gBlocks used were listed in Table S8 (Supporting Information).

##### RNA Preparation and RT‐PCR Analysis

Total RNAs were extracted from cells using TRIazol (Invitrogen, 15 596 018). Reverse transcription was performed with 1 µg of RNA using the Tetro cDNA synthesis kit (Bioline, BIO‐65043). Real‐time PCR was performed using DyNAmo Flash SYBR Green qPCR mix on a StepOnePlus system (Applied Biosciences) and normalized to *β*‐actin. The primers used for PCR are listed in Table S8 (Supporting Information).

##### Generation and Maintenance of Immunodeficient CD (Nur7) Mice

All animal housing conditions and surgical procedures were approved by and conducted according to the Institutional Animal Care and Use Committee of City of Hope. *ASPA* ^nur7/+^ (*ASPA* ^nur7^/J, 0 08607) and *Rag*2^−/−^ mice (B6(Cg)‐*Rag*2^tm1.1Cgn^/J, 0 08449) were purchased from the Jackson Laboratory. *ASPA* ^nur7/+^ mice were backcrossed with *Rag*2^−/−^ mice for four generations and screened for homozygosity of *ASPA* ^nur7/nur7^ and *Rag2* ^−/−^ mutations. The *ASPA* ^nur7/nur7^/*Rag2* ^−/−^ mice were called CD (Nur7) mice. The survival of the WT, Het, and CD (Nur7) mice, and the ASPA iNPC‐transplanted CD (Nur7) mice was monitored for 10 months. Animal death was not counted during the first 2 months for mice of all genotypes, because death resulted from pathology versus death resulted from events associated with fostering, cannibalization, and weaning occurred during this period was not differentiated.

##### Stereotaxic Transplantation

Postnatal day 1–4 (PND 1–4) mice were anesthetized on ice for 6–7 min and then placed onto a stereotaxic device. The ASPA iNPCs in suspension were transplanted with about 600 000 cells (in 1.5 µL) per site into six sites in the mouse brain bilaterally using a Hamiliton syringe with 33 gauge needle. The following coordinates, which were modified from a published study, ^[^ ^47^ ^]^ were used for transplantation: the corpus callosum (+3.0, ±1.6, −1.3), the subcortical (0.5, ±1.0, −2.5), and the brain stem (−1.6, ±0.8, −3.0). For pups with weight over 2 g and/or with head size obviously bigger than usual, slightly modified coordinates were used: the corpus callosum (+3.5, ±1.7, −1.4), the subcortical (0.5, ±1.0, −2.5), and the brain stem (−1.6, ±1.0, −3.1). All the coordinates are (A, L, V) with reference to Lambda. “A” stands for anteroposterior from midline, “L” stands for lateral from midline, and “V” stands for ventral from the surface of brain, respectively. The ASPA iOPCs were transplanted with about 60 000 cells (in 1.5 µL) per site into six sites per mouse brain using the same coordinates.

##### Immunohistochemistry

Immunohistochemistry was performed on PFA‐fixed tissues. Animals were deeply anesthetized and transcardially perfused with ice cold 0.9% saline followed by 4% PFA. Perfused brains were removed and postfixed in 4% PFA, then cryoprotected with 30% sucrose. Cryoprotected brains were flash frozen and stored at −20 °C. Then the brains were serially cryosectioned at sagittal planes. Specifically, slides were first labeled. Serial sections were collected onto labeled slides with one section per slide, until all slides were used for collection. Repeat the procedure until all sections from a brain were collected. For immunohistochemistry analysis, brain sections were permeabilized in PBST for 2 × 10 min, blocked with 5% donkey serum in PBST for 1 h at RT. Sections were then incubated with primary antibodies (Table S7, Supporting Information) at 4 °C for overnight. Following primary antibody incubation and washes, sections were incubated with secondary antibodies at RT for 2 h, washed with 1 x PBS, counterstained with Dapi, and mounted with the mounting medium. Cell fate and proliferation status were assessed by double immunostaining using the antihuman nuclear antigen (hNA) together with antibodies against PAX6, NeuN, SOX9, OLIG2, or Ki67. Confocal microscopy was performed on a Zeiss LSM 700 microscope (Zeiss), and the resulting images were analyzed with Zen 2.3 lite software (Zeiss). For quantification, images of transplanted cells in all three targeting sites including the corpus callosum, the subcortical and the brain stem regions were taken. Total human cells and double positive cells were counted for each brain. Three brains were analyzed in each group. Tiled whole section sagittal images were taken using Nikon Ti‐2 and dot maps were made using Photoshop CS4 based on the hNu^+^ signal from the titled whole section sagittal images.

##### NAA Level and ASPA Activity Measurement in Brain Tissues

Aqueous metabolites were extracted from mouse brains using the method of perchloric acid (PCA, Sigma, 244 252) as described.^[^ ^48^ ^]^ Briefly, mouse brains were rapidly chopped into small pieces, mixed well and divided into aliquots. Two aliquots were placed into two 1.5 mL Eppendorf tubes. Brain tissues in one tube were subjected to PCA extraction directly, while tissues in another tube were incubated at 37 °C for 1 h followed by PAC extraction. 6% ice‐cold PCA was added into each tube at 5 mL g^−1^ of wet brain tissues, followed by vortexing for 30 s. The samples were incubated on ice for additional 10 min. The mixture was centrifuged at 12 000 × *g* for 10 min at 4 °C. The supernatant was transferred into a new tube, neutralized with 2 m K_2_CO_3_, and placed on ice with lids open to allow CO_2_ to escape. Each sample was incubated on ice for 30 min to precipitate the potassium perchlorate salt. Supernatant was collected and pH was adjusted. Samples were centrifuged at 12 000 × *g* for 10 min at 4 °C. Supernatant was transferred to Eppendorf tubes and frozen on dry ice. Samples were then subjected to NMR analysis at the NMR Core Facility of City of Hope. The ASPA activity was calculated using the difference of NAA levels before and after 1 h incubation at 37 °C, and expressed as decreased NAA level in nmol per gram of brain tissue per hour.

##### Hematoxylin and Eosin Staining and Vacuolation Analysis

A one‐in‐six series of whole brain slides were stained with H&E at the Pathology Core of City of Hope. The whole slide was scanned under Nanozoomer HT (Hamamatsu Photonics, Japan) at the Light Microscopy Core of City of Hope. The surface area of the vacuolated brain regions and the intact brain regions was measured using Image‐Pro Premier 9.2 for all sections. The percent vacuolation = [the area of vacuolated brain region/(the area of vacuolated brain region + the area of intact brain region)] × 100. All sections from one representative slide of each brain were analyzed and at least three brains were analyzed for each mouse group.

##### EM and G‐Ratio Analysis of Myelin Sheaths

Mice were deeply anesthetized with isoflurane, and perfused with 0.9% saline followed by 0.1 m Millonig's buffer containing 4% PFA and 2.5% glutaraldehyde. Brain tissues were dissected and postfixed in the same fixative overnight. We followed a heavy metal staining protocol developed by Dr. Mark Ellisman's group.^[^ ^49^ ^]^ Target tissues were cut into about150 µm vibratome sections using a Leica VT 1000S vibratome. The subcortical white matter of the brain was microdissected and embedded in Durcupan ACM resin (Electron Microscopy Sciences). Ultrathin sections were cut using a Leica Ultracut UCT ultramicrotome and picked onto EM grids. Transmission electron microscopy was performed on an FEI Tecnai 12 transmission electron microscope equipped with a Gatan Ultrascan 2K CCD camera at the EM Core Facility of City of Hope. Three to four images were randomly taken for each sample in the subcortical region (3 images for the HET and the transplanted mice, respectively, and four images for CD (Nur7) mice). The inner axonal diameter and the total outer diameter of total 15 myelin sheathes in the brain of the Het and the transplanted mice, respectively, and 17 myelin sheathes in the brain of the CD (Nur7) mice were measured using Image‐Pro Premier 9.2. The g‐ratio is the ratio of the inner axonal diameter to the total outer diameter. The abnormal myelin sheaths were further identified based on the layer structure of the myelin sheaths which exhibited substantial difference between the Het and the CD (Nur7) mice.

##### Rotarod Test

The motor performance of the ASPA iNPC‐transplanted mice was evaluated using a rotarod treadmill (Rotamex, Columbus Instruments) as described.^[^ ^17^ ^]^ Mice were tested for the latency on the rod when the rod was rotating at the accelerating speed (2–65 rpm) in a 2 min trial session. Each mouse was monitored for the latency 4 times per test. At least 6 mice for each group were tested.

##### Grip Strength Test

The forelimb strength of the transplanted mice was measured using a grip strength meter (BIO‐GS3, Bioseb) to detect motor coordination and motor function. Mouse was allowed to grip a metal grid tightly. The grip strength of the mouse was recorded by gently pulling the tail of the mouse backward until release. Four sequential measurements were performed, and the average strength was calculated. At least 6 mice for each group were tested.

##### Mycoplasma Test

All cell culture products including iPSCs, iNPCs, and iOPCs were checked for potential mycoplasma contamination using MycoAlert PLUS Mycoplasma Detection Kit (Lonza). 500 µL culture medium was harvested from each cell line and centrifuged at 200 × *g* for 5 min to eliminate cell debris. 100 µL medium was used for each reaction and duplicate reactions were run for each sample. The result was determined by luminescence reading according to the established SOP. All cellular products used in this study were mycoplasma negative.

##### Statistical Analyses

Data are shown as means ± SE as specified in the figure legends and analyzed with GraphPad Prism 8 (San Diego, CA) and KaleidaGraph 4.0 (Reading, PA). The number of mice analyzed per treatment group is indicated as “*n*” in the corresponding figure legends. No exclusion criteria were applied. Animals were assigned randomly to treatment groups. The study was not blinded. Student's *t*‐test (two tailed), Log‐rank test, and One‐Way ANOVA followed by Dunnett's multiple comparisons test or Tukey's multiple comparisons test were used for statistical analysis as reported in each figure legend. *P* < 0.05 was considered statistically significant. *P < 0.05, **P < 0.01, and ***P < 0.001.

## Conflict of Interest

The authors declare no conflict of interest.

## Supporting information

Supporting Information
