# Supplementary material for: Cell‐Based Therapy for Canavan Disease Using Human iPSC‐Derived NPCs and OPCs
Source: Adv Sci (Weinh). 2020 Oct 29;7(23):2002155. doi: 10.1002/advs.202002155 (PMC7709977; doi:10.1002/advs.202002155)
Supplement: Supplementary file 1 — Supporting Information [file ADVS-7-2002155-s001.pdf]

© 2020 Wiley-VCH GmbH

ADVANCED SCIENCE
Open Access

Supporting Information

for *Adv. Sci.,* DOI: 10.1002/advs.202002155

Cell-Based Therapy for Canavan Disease Using Human iPSC-Derived NPCs and OPCs

*Lizhao Feng, Jianfei Chao, E Tian, Li Li, Peng Ye, Mi Zhang, Xianwei Chen, Qi Cui, Guihua Sun, Tao Zhou, Gerardo Felix, Yue Qin, Wendong Li, Edward David Meza, Jeremy Klein, Lucy Ghoda, Weidong Hu, Yonglun Luo, Wei Dang, David Hsu, Joseph Gold, Steven A. Goldman, Reuben Matalon, and Yanhong Shi**

Supporting Information

Cell-based therapy for Canavan disease using human iPSC-derived NPCs and OPCs

Lizhao Feng[1,], Jianfei Chao[1], E Tian[1], Li Li[1], Peng Ye, Mi Zhang, Xianwei Chen, Qi Cui, Guihua Sun, Tao Zhou, Geraldo Felix, Yue Qin, Wendong Li, Edward David Meza, Jeremy Klein, Lucy Ghoda, Weidong Hu, Yonglun Luo, Wei Dang, David Hsu, Joseph Gold, Steven A. Goldman, Reuben Matalon, Yanhong Shi[,]*

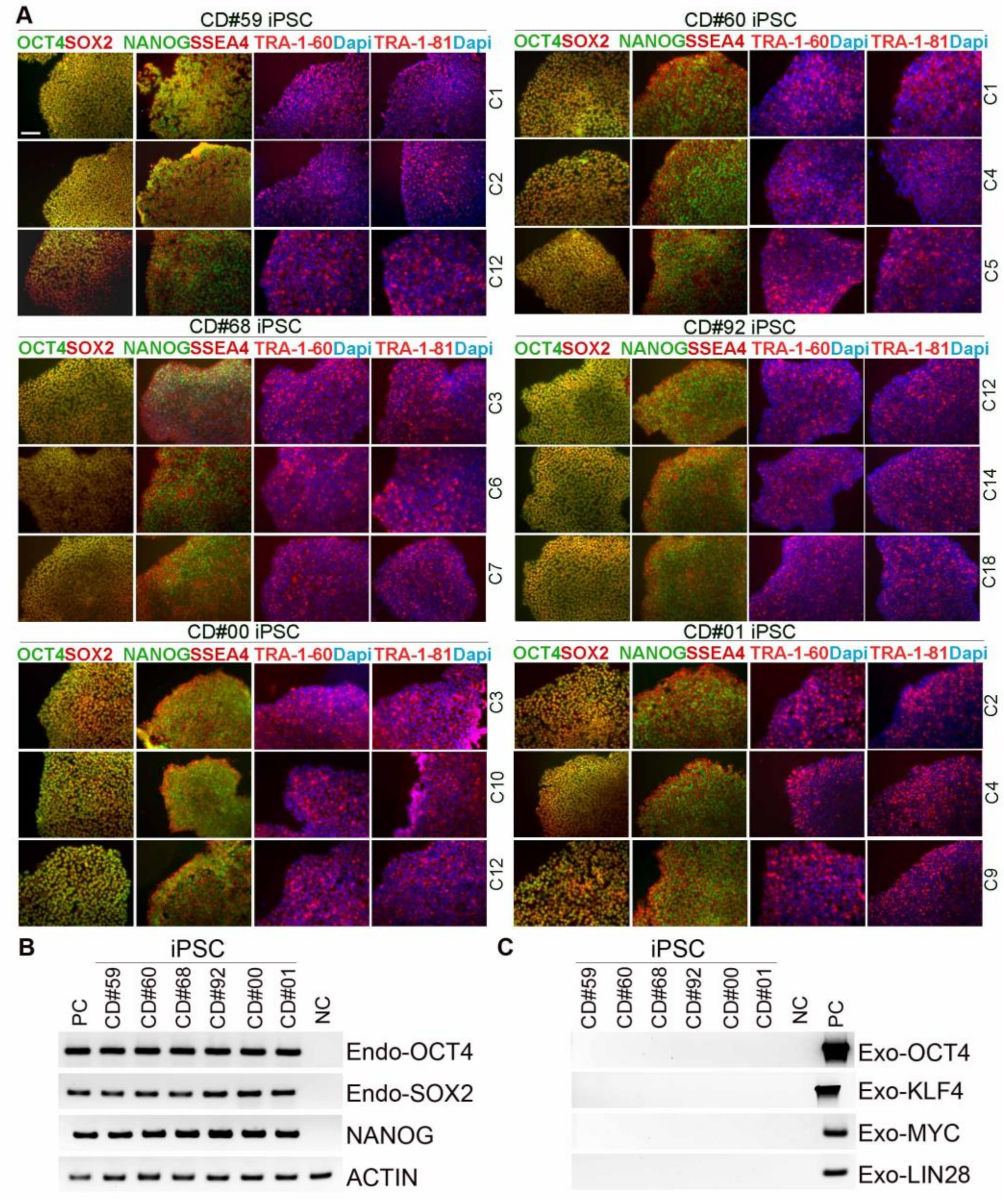

Figure S1. Characterization of CD iPSCs, related to Figure 1. (A) Expression of human pluripotency factors OCT4 and NANOG and the human ESC cell surface markers SSEA4, TRA-1-60 and TRA-1-81 in CD iPSCs. Three clones of CD iPSCs derived from each CD patient were included. Scale bar: 100 µm. (B) Expression of pluripotency factors in CD iPSCs. RT-PCR analysis of endogenous OCT4, SOX2, and NANOG expression in CD iPSCs. Human H9 ESCs were included as the positive control (PC), and fibroblast cells were included as the negative control (NC). ACTIN was included as a loading control. (C) Lack of residual reprogramming factors in CD iPSCs. PCR was performed to determine if there are any residual reprogramming factors in CD iPSCs. Fibroblast cells were included as the negative control (NC). The plasmid DNAs expressing individual reprogramming factors were included as the positive control (PC).

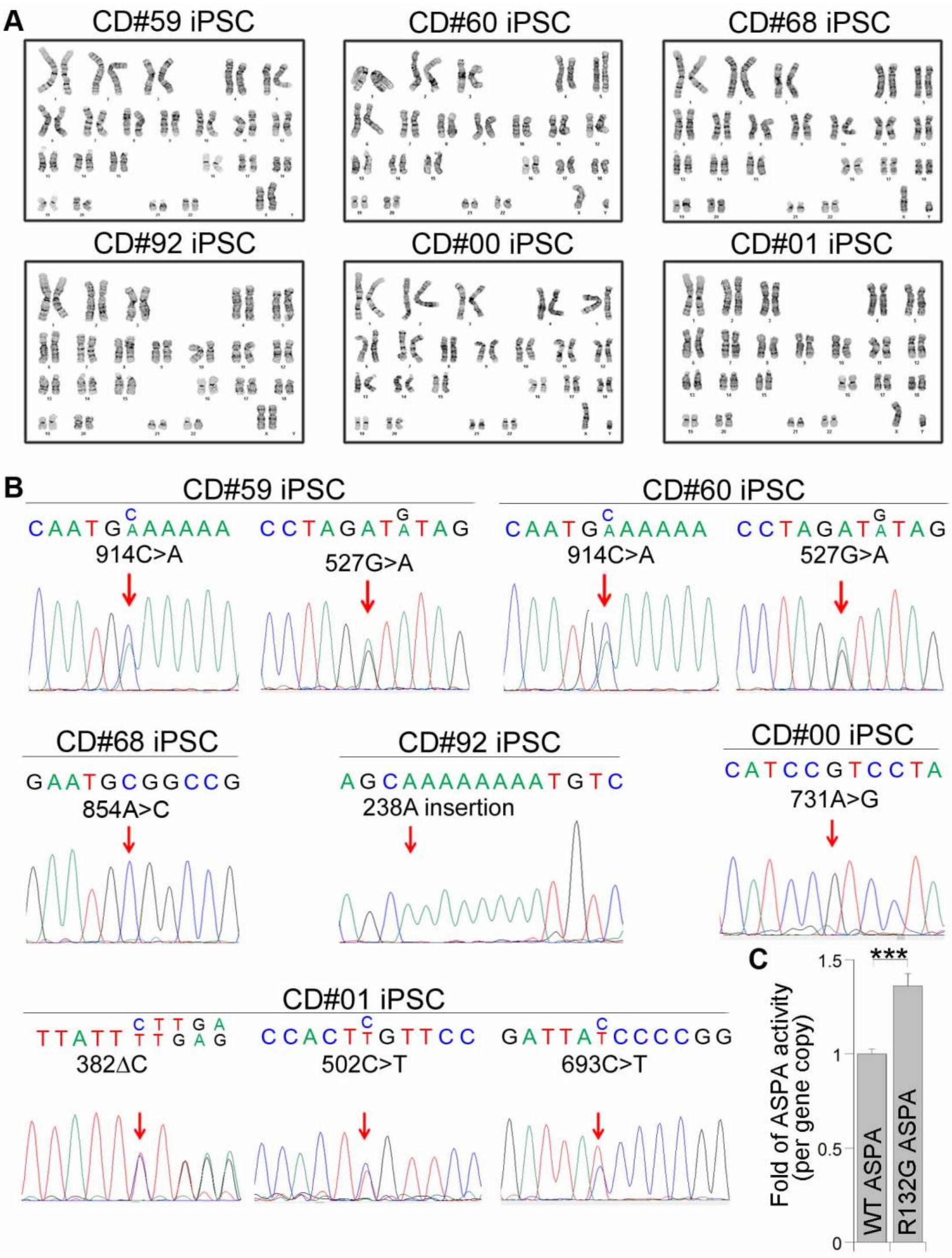

Figure S2. CD iPSCs exhibit normal karyotype and ASPA mutations, related to Figure 1. (A) CD iPSCs exhibit normal karyotype as reveal by G-banding. (B) CD iPSCs contained patient-specific *ASPA* mutations as revealed by Sanger sequencing. The arrows indicate mutation sites. (C) The R132G ASPA exhibits mildly increased ASPA activity per gene copy, compared to the wild type (WT) ASPA. The fold change is relative to the WT ASPA-transfected cells. ***p<0.001 by Student's t-test.

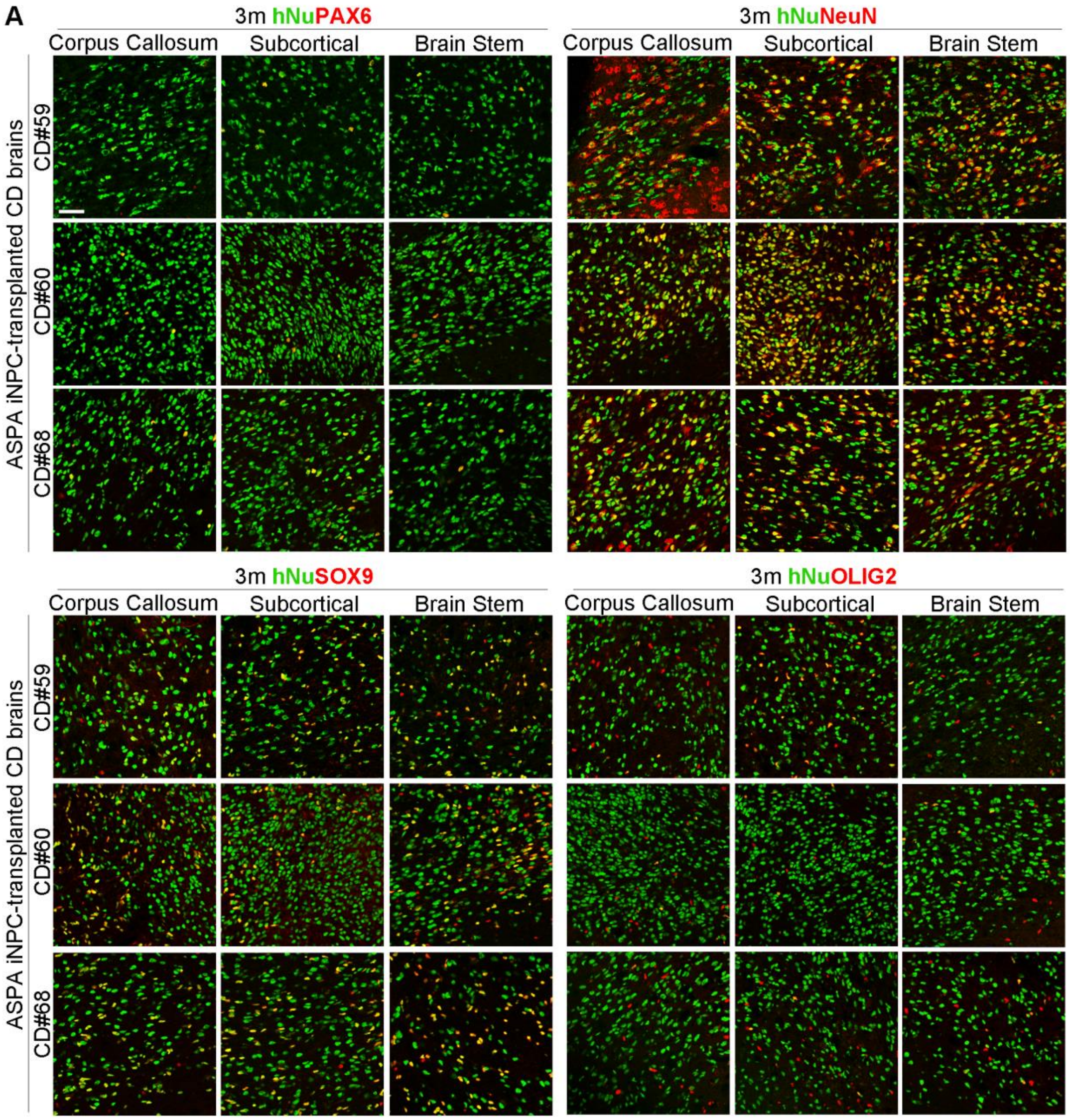
A
3m hNuPAX6
3m hNuNeuN
3m hNuSOX9
3m hNuOLIG2
Corpus Callosum
Subcortical
Brain Stem
ASPA iNPC-transplanted CD brains
CD#59
CD#60
CD#68

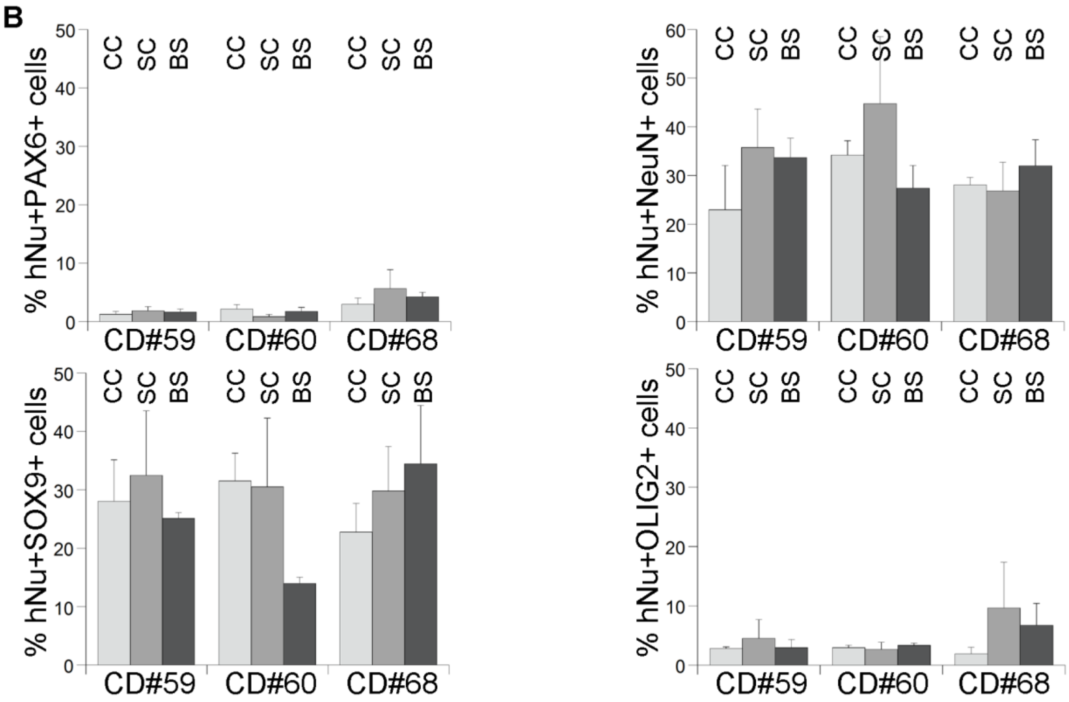

Figure S3. The cell fate of the ASPA iNPCs in different regions of transplanted CD (Nur7) mouse brains, related to Figure 2. (A) The ASPA iNPCs were transplanted into CD (Nur7) mouse brains. Three months (3m) after transplantation, the mouse brains were harvested and immunostained for hNu the NPC marker PAX6, the neuronal marker NeuN, the astrocyte marker SOX9, and the oligodendroglial lineage marker OLIG2, respectively. The ASPA iNPCs gave rise to neurons, astrocytes, and oligodendroglial lineage cells in the CD#59 ASPA iNPC, CD#60 ASPA iNPC and CD#68 ASPA iNPC -transplanted CD (Nur7) mouse brains. Scale bar: 50 µm. (B) The percentage of hNu^{+} and the neural lineage $marker^{+}$ cells in the different regions of transplanted brains. n=3 mice for each group.

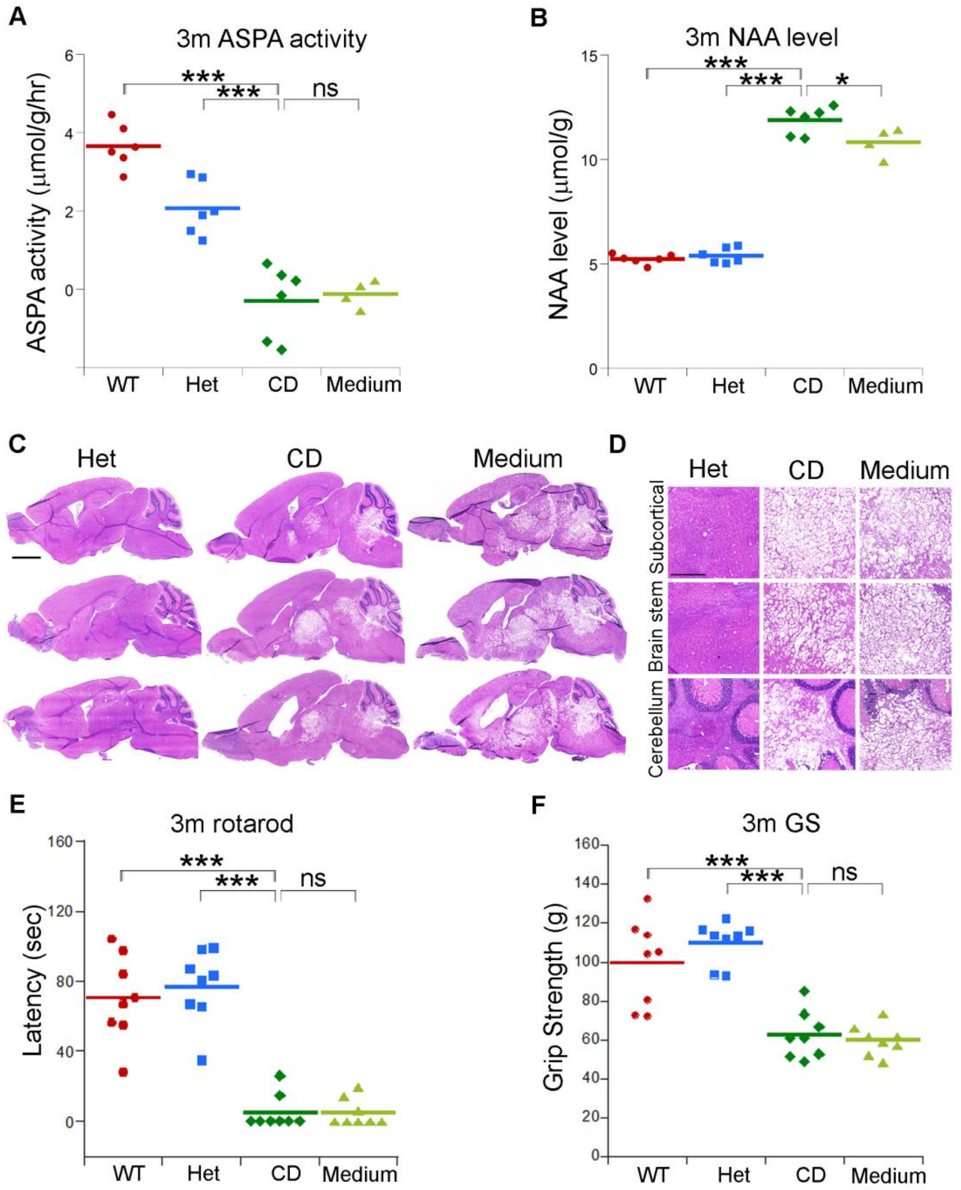

Figure S4. Medium-treated CD (Nur7) mice exhibit deficits similar to the un-transplanted CD (Nur7) control mice. The medium for ASPA iNPCs was injected into CD (Nur7) mouse brains using the same coordinates and procedure as for cell transplantation and the treated mice were analyzed three months post-treatment. (A, B) Low ASPA activity (A) and high NAA level (B) in medium-treated CD (Nur7) mouse brains three months after transplantation. The ASPA activity was expressed as reduced NAA level per gram of brain tissue in an hour (µmol/g/hr). The data for the WT, Het and CD (Nur7) mice from Figure 2E and 2F were included here as controls. Each dot represents the result from an individual mouse for panels A & B. n=6 mice for WT, Het, and CD (Nur7) mice, respectively, and 4 for medium-treated mice. (C, D) Vacuolation in brains of medium-treated CD (Nur7) mice as revealed by H&E staining. The data for the WT, Het and CD (Nur7) mice from Figure 3A and 3B were included here as controls. Scale bar: 2,000 µm for C and 500 µm for D. (E, F) Deficit of motor function in medium-treated CD (Nur7) mice three months after transplantation as revealed by rotarod (E) or grip strength (GS, F) test. Each dot represents the result from an individual mouse for panels E & F. The data for the WT, Het and CD (Nur7) mice from Figure 4D and 4E were included here as controls. n=8 mice for WT, Het, CD (Nur7) mice and medium-treated CD (Nur7) mouse.

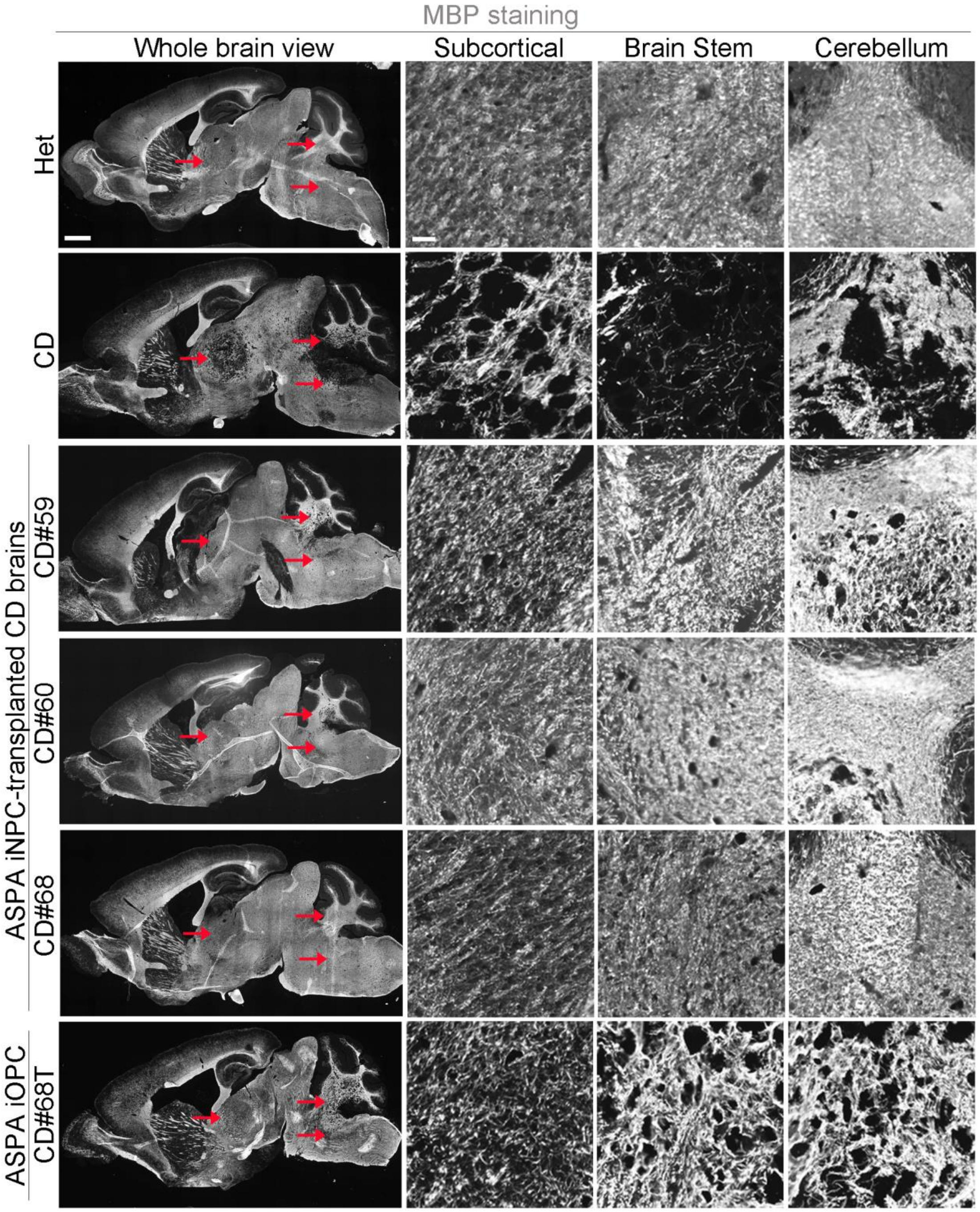

Figure S5. Myelination in the ASPA iNPCs and ASPA iOPC-transplanted CD (Nur7) mouse brains. Three months after transplantation, the mouse brains were harvested and immunostained for the myelination marker MBP. The whole brain sagittal sections are shown in the left panels. The red arrows indicate areas in which the myelination extent is different in the CD (Nur7) mice, compared to that in the Het or transplanted mice. Enlarged images of the subcortical white matter, the brain stem and the cerebellum are shown in the right panels. Scale bar: 1,000 μm for whole brain sagittal section images, and 50 μm for enlarged images.

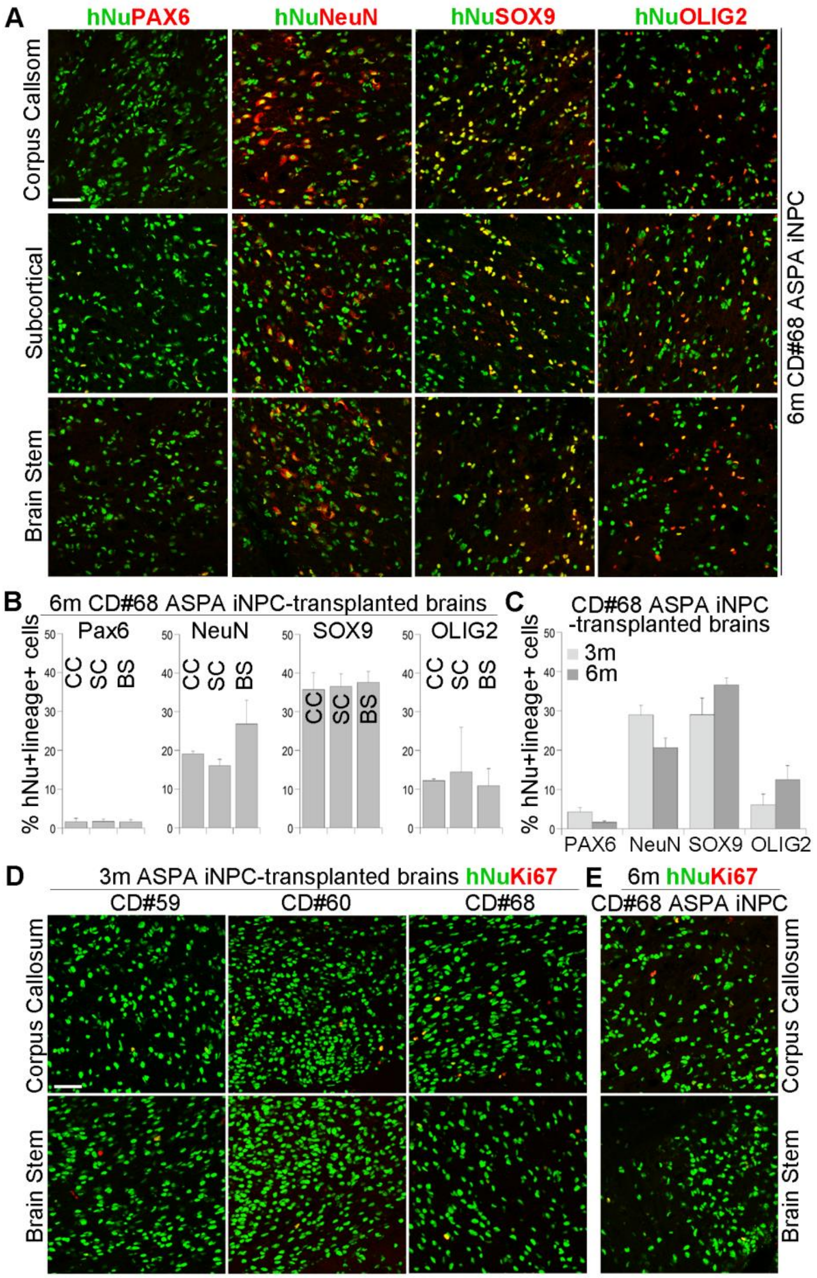

Figure S6. The cell fate of the ASPA iNPCs in transplanted CD (Nur7) mouse brains six months after transplantation, related to Figure 5. (A) The ASPA iNPCs gave rise to neurons, astrocytes, and oligodendroglial lineage cells in the CD#68 ASPA iNPC-transplanted CD (Nur7) mouse brains. Six months after transplantation, the ASPA iNPC-transplanted brains were immunostained for hNu and the NPC marker PAX6, the neuronal marker NeuN, the astrocyte marker SOX9, and the oligodendroglial lineage marker OLIG2, respectively. Scale bar: 50 µm. (B) The percentage of hNu^+ and the neural lineage $marker^+$ cells in the different regions of transplanted brains. n=3 mice for each marker. Error bars are SE of the mean. (C) The percentage of hNu^+ and the neural lineage $marker^+$ cells in the CD#68 ASPA iNPC-transplanted CD (Nur7) mouse brains three and six months after transplantation. The 3-month quantification data from Figure 2D was included here for comparison. n= 9 fields from 3 mice for each group. Scale bar: 50 µm. (D, E) Low mitotic index in ASPA iNPC-transplanted CD (Nur7) mouse brains as revealed by hNu and Ki67 co-staining three (D) or six months (E) after transplantation. The images from the corpus callosum and the brain stem are shown. Scale bar: 50 µm.

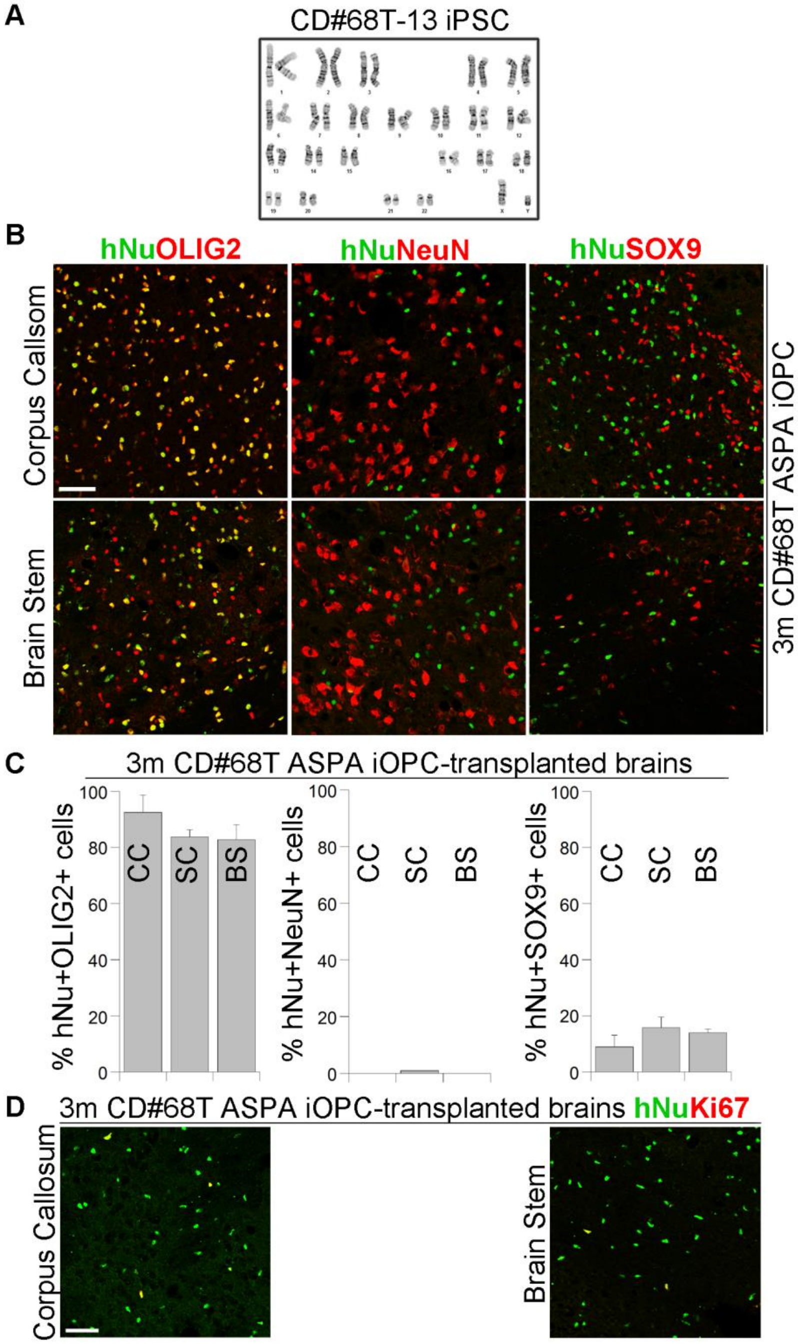

Figure S7. Characterization of ASPA iOPCs, related to Figure 6. (A) The CD#68T-13 ASPA iOPCs exhibited normal karyotype. (B) Co-staining of the transplanted CD (Nur7) mouse brains for human nuclear antigen hNu and the oligodendroglial lineage marker OLIG2, the neuronal marker NeuN, or the astrocyte marker SOX9, respectively. The corpus callosum and the brain stem regions were shown. (C) The percentage of the $hNu^{+}NeuN^{+}$, $hNu^{+}SOX9^{+}$, and $hNu^{+}OLIG2^{+}$ population in the different regions of ASPA iOPC-transplanted (Nur7) mouse brains. n=3 mice for each group. (D) The ASPA iOPCs showed low mitotic index in transplanted mouse brains as revealed by hNu and Ki67 co-staining. The corpus callosum and the brain stem regions are shown. Scale bar: 50 µm. Error bars are SE of the mean.

Table S1 Characterization of CD iPSCs		
**Tests**	**Method**	**Specification**
Sterility	USP Sterility	No growth
Mycoplasma	Luminescence assay	Negative
Karyotype	G-banding	Normal
STR assay	PCR	100% identity
Purity	Flow cytometry	>90% $SSEA4^{+}$ cells
Residual exogenous reprogramming factors	PCR	Not detectable

Table S2 CD iPSCs Exhibit the Same STR Pattern as Parental Fibroblasts

Locus /lines	CD59		CD60		CD68		CD92		CD00		CD01	
	Fib	iPSC	Fib	iPSC	Fib	iPSC	Fib	iPSC	Fib	iPSC	Fib	iPSC
AMEL	X	X	X, Y	X, Y	X, Y	X, Y	X	X	X, Y	X, Y	X, Y	X, Y
CSF1PO	11, 12	11, 12	11	11	11	11	10, 12	10, 12	10, 11	10, 11	12, 13	12, 13
D13S317	8, 11	8, 11	8, 11	8, 11	11, 12	11, 12	12	12	11, 13	11, 13	11, 12	11, 12
D16S539	11, 12	11, 12	12	12	10, 13	10, 13	12	12	12	12	9, 10	9, 10
D21S11	30, 31.2	30, 31.2	30, 31.2	30, 31.2	29, 31.2	29, 31.2	29, 30	29, 30	30	30	30, 31	30, 31
D5S818	10, 12	10, 12	11, 12	11, 12	10, 12	10, 12	9, 12	9, 12	12, 13	12, 13	12	12
D7S820	9, 11	9, 11	11	11	9, 12	9, 12	10, 11	10, 11	11	11	10	10
TH01	9.3	9.3	6, 9.3	6, 9.3	9, 9.3	9, 9.3	7	7	6, 7	6, 7	7, 9.3	7, 9.3
TPOX	8, 11	8, 11	8, 11	8, 11	9, 11	9, 11	8	8	8	8	8, 12	8, 12
vWA	14, 17	14, 17	14, 18	14, 18	17, 18	17, 18	16,17	16,17	18	18	14, 17	14, 17

Note: If both alleles at a locus have the same STR genotype, only one X or number is shown.

Table S3 Flow Cytometry Analysis of CD iPSCs								
**% Cells**	**HEK 293T**	**H9 ESC**	**CD59 iPSC**	**CD60 iPSC**	**CD68 iPSC**	**CD92 iPSC**	**CD00 iPSC**	**CD01 iPSC**
% OCT4$^+$ cells	-0.008	98.95	98.26	98.47	98.08	97.80	96.99	98.06
% SSEA4$^+$ cells	0.011	98.96	98.86	93.26	99.29	99.83	99.55	97.64

Table S4 ASPA iNPCs Exhibit the Same STR Pattern as Parental Fibroblasts												
**Locus /lines**	**CD59**		**CD60**		**CD68**		**CD92**		**CD00**		**CD01**	
	**Fib**	**ASPA iNPC**	**Fib**	**ASPA iNPC**	**Fib**	**ASPA iNPC**	**Fib**	**ASPA iNPC**	**Fib**	**ASPA iNPC**	**Fib**	**ASPA iNPC**
AMEL	X	X	X, Y	X, Y	X, Y	X, Y	X	X	X, Y	X, Y	X, Y	X, Y
CSF1PO	11, 12	11, 12	11	11	11	11	10, 12	10, 12	10, 11	10, 11	12, 13	12, 13
D13S317	8, 11	8, 11	8, 11	8, 11	11, 12	11, 12	12	12	11, 13	11, 13	11, 12	11, 12
D16S539	11, 12	11, 12	12	12	10, 13	10, 13	12	12	12	12	9, 10	9, 10
D21S11	30, 31.2	30, 31.2	30, 31.2	30, 31.2	29, 31.2	29, 31.2	29, 30	29, 30	30	30	30, 31	30, 31
D5S818	10, 12	10, 12	11, 12	11, 12	10, 12	10, 12	9, 12	9, 12	12, 13	12, 13	12	12
D7S820	9, 11	9, 11	11	11	9, 12	9, 12	10, 11	10, 11	11	11	10	10
TH01	9.3	9.3	6, 9.3	6, 9.3	9, 9.3	9, 9.3	7	7	6, 7	6, 7	7, 9.3	7, 9.3
TPOX	8, 11	8, 11	8, 11	8, 11	9, 11	9, 11	8	8	8	8	8, 12	8, 12
vWA	14, 17	14, 17	14, 18	14, 18	17, 18	17, 18	16,17	16,17	18	18	14, 17	14, 17

Note: If both alleles at a locus have the same STR genotype, only one X or number is shown.

Table S5 No mutation was detected in the top potential off-target sites as revealed by WGS						
**ID**	**Chromosome**	**Position 1**	**Position 2**	**Sequence 1**	**Sequence 2**	**Mutation**
1	chr19	55627106	55627146	TTTTCTGTCACCAATCCT	TTATCTGTCCCCTCCACC	No
2	chr19	55627106	55627148	TTTTCTGTCACCAATCCT	TTTTATCTGTCCCCTCCA	No
3	chr3	33655954	33655990	ATTTCTGTCAAAAATCCT	TTGTCTTTCACTAATACT	No
4	chr22	51139502	51139538	CTCCCCCCACCCCCCAAA	TTTTCTGTCCCCACTCCA	No
5	chr5	126016079	126016113	TACTCCCCACCCCACAGA	TCCCCTGCATCCAACAGT	No
6	chr17	64789835	64789872	TCCACCCTACCTCCCAGC	TACCCTCCTCCCCACAGT	No
7	chr3	51729812	51729854	TTTTTTGCCAACACTCCT	CCACCACCACCCCACCCT	No
8	chr20	9514366	9514406	TCCCCTCCACCCCAGTTC	TCCCCTCAACCCAAAACT	No
9	chr9	135125487	135125528	TCCCCTCCTGCCCACAGA	TCCCCTACCCCCCAAACA	No
10	chr17	16923065	16923106	TTCTCACCACCCCACACT	TCCCTTCCCACCCAAAGT	No
11	chr5	177973159	177973194	TCCCCTCCACCTCAAACA	CCCCCGCCACCCCATATT	No
12	chr20	30459310	30459347	TCACCCCCACCCCTCAAT	CTCTCTATCCACAATCCC	No
13	chr19	1854107	1854149	TCCACTCCACCCCCCCAA	TCCTCTCCACCACCCCCT	No
14	chr22	51139503	51139538	TCCCCCCACCCCCCAAAA	TTTTCTGTCCCCACTCCA	No
15	chr22	51139504	51139538	CCCCCCACCCCCCAAAAA	TTTTCTGTCCCCACTCCA	No
16	chrX	31479737	31479778	TCTTCCATCACTAATTCT	TACCCTCCACCCTACCAT	No
17	chr8	66106608	66106646	TTTTCTGTAACCACTCCT	TGTTCTGACACTTTTCCC	No
18	chr10	71712424	71712459	CCCCCTCCCACCCACCTT	TCCTCCCCACCCCCCAGG	No
19	chr10	71712418	71712459	TCCCCTCCCCCTCCCACC	TCCTCCCCACCCCCCAGG	No
20	chr19	35716209	35716246	TCCTCGCCACCCCCCACG	CCTCCTCCACCCCACTGT	No
21	chr1	23205692	23205728	CCACCTCCACCACACACA	TCTCATCCAACCCACAGG	No
22	chr18	66577716	66577755	TTTTATATCACCAACCCC	TTTTCTGTGATCAATTAT	No
23	chr12	23734089	23734128	TTCTCTCTCCCCACTCCT	TTCTCTATTAAAACTCCT	No
24	chr6	167286954	167286986	CCGTCAGTCACCCCTCCT	CCCACTCCACCCCACTGT	No
25	chr22	51139505	51139538	CCCCCACCCCCCAAAAAA	TTTTCTGTCCCCACTCCA	No
26	chr3	151635330	151635367	TGCCCCCCAACCCACCAA	TTTTCTTTCACCAATACC	No
27	chr20	25440058	25440099	ACACCTGCACCCCACATT	TTTTCTGTCTCAAACCAT	No
28	chr6	148392411	148392443	TCCCCTCATCCCCACATC	TCCCCAACATCACACACT	No
29	chr16	89289717	89289757	TTCTGTGGAACCAATACT	TTTGCCGTCACCAACCCT	No
30	chr11	57225403	57225434	CCCCCTCCCCCCAACCCT	TTTGCTGTCCCCACCCCA	No
31	chr1	109800575	109800608	CCCACTCCCCCCCACCCC	TCCCCACTCCCCCACAGC	No
32	chr1	227647992	227648030	TTTTCTATTAACAAAAAT	TCCCCTCCTCACCACTGT	No
33	chr20	19431018	19431059	TTTCATCTCCCCAGCCCT	CCTCCTCCTCCCCACAGT	No
34	chr8	145493633	145493673	TTTCCTCTCCCTAATCCT	TCCTCTCCTCCCCTCCCT	No
35	chr8	145324548	145324588	TTTCCTCTCCCTAATCCT	TCCTCTCCTCCCCTCCCT	No
36	chr1	240682937	240682971	TTTTCTGAAACCAATCCT	CTTTTTATCCCCAGTACA	No
37	chr6	101328557	101328598	ATTTCTTTCCCCAAATCT	TTTTCTGTTACAAAGCCT	No
38	chr7	81302717	81302749	TTTTCTTACAGCAACACT	TTTACTGTCACCACTACT	No
39	chr1	62776649	62776690	TATTCTGTCATCACTCCT	TCCCCTCCCACCCTTAAC	No
40	chr3	124702671	124702707	TCCCTTCCACCTCACCAA	TGTTCTGTACCCAACCCT	No
41	chr16	9446225	9446264	TTCTCTCCACACCACAGT	ATTGCTTACACCACTCCT	No
42	chr3	188629607	188629647	TCCCCACCACCCCAAAGA	TTTTGAATCAACACTCCC	No
43	chr16	85176136	85176173	TCCCCTCTCCCCCACAGT	CCCCCTCCACAGCCTCAT	No
44	chr1	23443971	23444002	CCCCCCCCCCCCCACAAT	CCCCCCCCGCCCCCCCCC	No
45	chr2	27902887	27902923	TCCCCCCAACCCTTCATT	CCCCTCCCACCCCCCAGT	No
46	chr3	1948778	1948810	TTCTCTGTCACCAGCCCA	TTTTACCTTAACAATCCT	No
47	chr5	165831361	165831403	TTTTCTATAACTCATATT	CCCCCTCCACCCCAAAAA	No
48	chr4	182910724	182910754	TTTCTTTTCACTATTCCT	TTTTACATCAACAATCCT	No
49	chr13	103511981	103512019	TTTTCTTTCTGCAACCAT	TCCACACCACCCTACAGT	No
50	chr16	11588172	11588209	CCCCCTTCACCCCACCCC	CCCCCCCCACACCACTGC	No

ID	Chromosome	Position 1	Position 2	Sequence 1	Sequence 2	Mutation
51	chr1	210512105	210512143	TATGCTGTCTCCATGCCT	TCTTCTGCCACCAGTCCT	No
52	chr2	26398304	26398335	ACCCCTCCAACCCTCAGC	CCCCCTCATCCTCCCAGT	No
53	chr5	127879161	127879199	CCACCTCCACCTAACAGT	TCTTCTGACACCATCCCA	No
54	chr15	66978743	66978773	TCCACTCCACCCCCTCTT	CCCCCTCAACCACCCAGC	No
55	chr2	172495090	172495127	TCCCATCTCACCCAAAAT	TTTTCCATCACCAATTCA	No
56	chr8	100340500	100340539	TTGTCTTCCACAAAACCT	CTTTCTATCAGCACTCCT	No
57	chr2	157188240	157188270	TCCCCTCCCCCACAATCT	TCCCCCCACCCCCACCAC	No
58	chr8	126808188	126808223	TTCTCTGTGCACAAGCCT	TCTTCTGTCACCTGTCCT	No
59	chr8	107823093	107823123	TCCCCACCCTCCCACATT	CTCTCTGGCACCCTTCCT	No
60	chr9	135721616	135721651	TATCCAGACACCCACCCT	CCCCATCCACCCCACACA	No
61	chr11	26039493	26039534	ACCTCTGCCTCCCATCCT	CCCCCTCCACCCCCCGAT	No
62	chr1	23751270	23751301	CCACCCCCACCCCCCCAC	ACCCCTCCCCCGCACAGT	No
63	chr5	152496951	152496988	ATTTCTTTCCCCCATACT	TTCCCTGCCACCAACCCC	No
64	chr5	59268073	59268112	CTTTCTCTCACCTATTCT	TACTCAGTATCCAATCCT	No
65	chr12	127662432	127662471	TCTCACCCACCCAACAGT	TGCCCTCCTACCCACAAC	No
66	chr7	133549428	133549458	TCTTCTGTCACTAAAACT	CTTTCTGGCACATATACT	No
67	chr3	151635331	151635367	GCCCCCCAACCCACCAAA	TTTTCTTTCACCAATACC	No
68	chr11	22605631	22605667	TCCCCCCCACCCCGCCAA	CCACCTCCACCACAAGGT	No
69	chr5	59756990	59757024	TTTCCCTTCAAACATCCA	CTTTCTGTCACCCATCAT	No
70	chr19	38858131	38858173	CCCCTCCAACCCCATAGT	TCTCCTGTCACCAAATCT	No
71	chr9	114125386	114125428	TCCCCCCCACCCCACCCA	TTTCTTCTCCCCTAACCT	No
72	chr21	36774097	36774136	TCCTTTCCCACCAATCCT	TTCCTTCCACCCCACTGC	No
73	chr6	75057747	75057788	TTTTCTGTCCCCAAACAG	TCCTCCCCACCACCAAAT	No
74	chr14	100472345	100472376	TCCCCCCCCCCCCCCACA	TCACCCCCAACACACACA	No
75	chr12	125339766	125339804	CCCCCTCCCCAGCACAGG	TTCCCTCCACTCCACTGT	No
76	chr16	89368527	89368566	CTCCCTCCACCCCACTCA	TCCCCTCCTTCCCCCAGA	No
77	chr1	202164560	202164600	CCCCCTCCACTCCAAAAG	TTTTCTCTTCCCACTCCC	No
78	chrX	151141431	151141469	TACCCCCTCCCAAACAGT	GCCCCTCCCCCCCCCACT	No
79	chr12	50475768	50475800	TCTGCTGCCACCAGTCAC	TCCCTTCCACCCCTCACT	No
80	chr14	57361405	57361444	TCCCCTCCAACCCAAACC	CTTTCATTCACAATCCCT	No
81	chr7	94322229	94322265	TCTTATTTCACTAAGACT	TTTCCTCTCACCAGTCCT	No
82	chr18	45316703	45316741	AGCCCTCCAACCCACAGT	TCTTTTCCTCCCCACAAT	No
83	chr5	134897098	134897136	TCCCTCCCACCACACAGC	ACTTCCCCACCCCACAGC	No
84	chr13	22412475	22412508	TCACCTCCCCCCAAAAAT	TCTTCACTCCCCAAAACT	No
85	chr7	106691643	106691681	TTTTCTGTCTTCCATCAA	TTTTCTTTAGCCCATCCT	No
86	chr9	9197758	9197799	TTTCCTGTCAACACTCCT	CATTCCTTCAAAAATCAT	No
87	chr18	73098165	73098199	TTTTCTCTCCTCTCTCCT	TTTTCTGGCATCATTCAT	No
88	chr1	19736944	19736978	TCCCCCCAAACACACACA	CCCCCACCACCCCCCACC	No
89	chr12	54204713	54204755	TTCCCTCTCTCTACTCCT	CTTTCTCTCCCCAATTCT	No
90	chr16	74524192	74524225	CTTTATCTCCCCAAGCAA	TTTTCTATCACTAAACCT	No
91	chr4	56586353	56586394	GTTTATGTCCCCAATCCC	ATTTATATCCACAATCAT	No
92	chr19	35716204	35716246	CCCCCTCCTCGCCACCCC	CCTCCTCCACCCCACTGT	No
93	chr15	46816195	46816234	TGTTCTGTAACCAATACT	TTTTCTTGCCCAGACCCT	No
94	chr3	49903243	49903280	CACCCCCCACCCCACTCC	TCCCACCCACGCCACACT	No
95	chr21	39941092	39941133	TCCCCCCCCACCCCCAGT	TATTCATTCAACAAACAT	No
96	chr10	113458996	113459030	TTTTCCCTTACCAATCTA	TTTTCCCTCACCAATCTA	No
97	chr20	30586833	30586874	CCCCCACCCCCCCACACA	TCCTCTCCCACCAATCAC	No
98	chr1	23443971	23444005	CCCCCCCCCCCCCACAAT	TCCCCCCCCCCGCCCCCC	No
99	chr4	75724475	75724505	TTCCCTCCTTCCCCCAAC	TTTCCTGTCACCATTTCT	No

Table S6 List of Plasmids		
**Plasmid Name**	**Vendor**	**CAT. #**
pCXLE-hSK	Addgene	Cat# 27078
pCXLE-hUL	Addgene	Cat# 27080
pCXLE-hOct3/4-shp53-F	Addgene	Cat# 27077
pCXWB-EBNA1	Addgene	Cat# 37624
hAAVS1 TALEN Right	Addgene	Cat# 52342
hAAVS1 TALEN Left	Addgene	Cat# 52341
AAVS1-CAG-hrGFP	Addgene	Cat# 52344

Table S7 List of Antibodies		
**Antibody Name**	**Vendor**	**CAT. #**
Rabbit monoclonal anti-NANOG	Cell Signaling	Cat# 4903
Mouse monoclonal anti-OCT4	Santa Cruz	Cat# sc-5279
Goat polyclonal anti-SOX2	Santa Cruz	Cat# sc-17320
Mouse monoclonal anti-SSEA4	Santa Cruz	Cat# sc-21704
Mouse monoclonal IgM anti-Tra-1-60 Santa Cruz	Santa Cruz	Cat# sc-21705
Mouse monoclonal IgM anti-Tra-1-81 Santa Cruz	Santa Cruz	Cat# sc-21706
Mouse monoclonal anti-NESTIN	Fisher (BD Biosciences)	Cat# 611659
Goat polyclonal anti-SOX1	R&D	Cat# AF3369
PE-conjugated anti-SSEA4	BD Biosciences	Cat# 560128
PE-conjugated mouse IgG3, isotype control (SSEA4 control)	BD Biosciences	Cat# 559926
APC-conjugated anti-CD133	BD Biosciences	Cat# 566596
APC-conjugated mouse IgG1, isotype control (CD133 control)	BD Biosciences	Cat# 554681
PE-conjugated anti-Oct3/4	BD Biosciences	Cat# 560186
PE-conjugated mouse IgG1, isotype control (Oct3/4 control)	BD Biosciences	Cat# 554680
Mouse monoclonal IgM anti-O4	Sigma-Aldrich	Cat# O7139
PE-conjugated anti-CD140a	BD Biosciences	Cat# 556002
PE-conjugated mouse IgG2a, isotype control (CD140a control)	BD Biosciences	Cat# 555574
APC-conjugated anti-CD19	ThermoFisher Scientific	Cat# MHCD1905

Mouse monoclonal anti-human nuclear antigen antibody [235-1], hNu	Abcam	Cat# Ab191181
Rabbit polyclonal anti-PAX6	Biolegend	Cat# 901301
Rabbit polyclonal anti-OLIG2	Millipore	Cat# AB9610
Rabbit polyclonal anti-GFAP	Agilent (Dako)	Cat# Z033429-2
Goat polyclonal anti-SOX9	R&D	Cat# AF3075
Goat polyclonal anti-SOX10	R&D	Cat# AF2864
Rabbit polyclonal anti-NEUN	GeneTex	Cat# GTX16208
Rabbit monoclonal anti-Ki67	ThermoFisher Scientific	Cat# RM-9106-S0

**Table S8 List of Primers**	
**Primer Name**	**Sequence**
ASPA-Exon1-Fwd	5’-CTCCACTCAAGGGAATTCTGT-3’
ASPA-Exon1-Rev	5’-ACTGCATGTACGGACATGAA-3’
ASPA-Exon2-Fwd	5’-AGATTTGGCGACTGGTTCT-3’
ASPA-Exon2-Rev	5’-TGCACCTTCCCTCATAACTG-3’
ASPA-Exon3-Fwd	5’-ACTCTGTTGAAGCAAAGAGA-3’
ASPA-Exon3-Rev	5’-CAGAGCAAGACTCTGTCTCA-3’
ASPA-Exon4-Fwd	5’-TTCCATGATGCTACATGGTT-3’
ASPA-Exon4-Rev	5’-GCAAATCTGACCCAGGTTCCA-3’
ASPA-Exon5-Fwd	5’-TGTTCTCGAACTCCTGACCT-3’
ASPA-Exon5-Rev	5’-GCGAAGTGCTGTATGAGCTA-3’
ASPA-Exon6-Fwd	5’-GATCAAGACTGGAAACCAC-3’
ASPA-Exon6-Rev	5’-GAAGTGTAGTAAGGCAAAGC-3’
Endo-*OCT4*-Fwd	5’-CCTCACTTCACTGCACTGTA-3’
Endo-*OCT4*-Rev	5’-CAGGTTTTCTTTCCCTAGCT-3’
Endo-*SOX2*-Fwd	5’-CCCAGCAGACTTCACATGT-3’
Endo-*SOX2*-Rev	5’-CCTCCCATTTCCCTCGTTTT-3’
Endo-*NANOG*-Fwd	5’-GAATCTTCACCTATGCCTGTG-3’
Endo-*NANOG*-Rev	5’-ATCATTGAGTACACACAGCTG-3’
Exo-*OCT4*-Fwd	5’-CTCTAGAGCCTCTGCTAACCA-3’

Exo-*OCT4*-Rev	5’-TGTGCATAGTCGCTGCTTGAT-3’
Exo-*KLF4*-Fwd	5’-GCTCCCATCTTTCTCCACGTT-3’
Exo-*KLF4*-Rev	5’-GAAGCTTGAATTCCTGCAGGCA-3’
Exo-*LIN28*-Fwd	5’-AGAGCATCAGCCATATGGTAG-3’
Exo-*LIN28*-Rev	5’-GAAGCTTGAATTCCTGCAGGCA-3’
Exo-*L-MYC*-Fwd	5’-CTCTAGAGCCTCTGCTAACCA-3’
Exo-*L-MYC*-Rev	5’-TCGAATTTCTTCCAGATGTCC-3’
ASPA-Fwd	5’-CGGAATTCATGACTTCTTGTCAC-3’
ASPA--Rev	5’-GGACTAGTCTAATGTAAACAGCAG-3’
ACTIN-Fwd	5’-CCGCAAAGACCTGTACGCCAAC-3’
ACTIN-Rev	5’-CCAGGGCAGTGATCTCCTTCTG-3’
SOX1-Fwd	5’-AATACTGGAGACGAACGCCG-3’
SOX1-Rev	5’-AGTGCTTGGACCTGCCTTAC-3’
PAX6-Fwd	5’-GTGTCCAACGGATGTGTGAG-3’
PAX6-Rev	5’-CTAGCCAGGTTGCGAAGAAC-3’
AAVS1-Fwd	5’-CTCTAACGCTGCCGTCTCTC-3’
AAVS1-Rev	5’-GCTTCTCCTCTTGGGAAGTG-3’
ASPA-Rev	5’-AGCTCATTCCCATGGGTTCC-3’
PBS/psi-Fwd	5’-ACTTGAAAGCGAAAGGGAAAC-3’
PBS/psi-Rev	5’-TTTGGCGTACTCACCAGTC-3’
PBS/psi-TaqMan probe	5’-FAM-AGCTCTCTCGACGCAGGACTCGGC-TAMRA-3’
Albumin-Fwd	5’-TGAAACATACGTTCCCAAAGAGTTT-3’
Albumin-Rev	5’-CTCTCCTTCTCAGAAAGTGTGCATAT-3’
Albumin-TaqMan probe	5’-FAM-TGCTGAAACATTCACCTTCCATGCAGA-TAMRA-3’
PBS/psi-gBlock	5’-TCTAGCAGTGGCGCCCGAACAGGGACTTGAAAGCGAAAGGGAAACCAGAGGAGCTCTCTCGACGCAGGACTCGGCTTGCTGAAGCGCGCACGGCAAGAGGCGAGGGGCGGCGACTGGTGAGTACGCCAAAAATTTTGACTAGCGGAGGCT-3’
Albumin-gBlock	5’-CATGGCGGCCGCGGGAATTCGATTTGAAACATACGTTCCCAAAGAGTTTAATGCTGAAACATTCACCTTCCATGCAGATATATGCACACTTTCTGAGAAGGAGAGAATCACTAGTGAATTCGCGG-3’
REX1	Hs01938187_s1 (Thermo Fisher Cat# 4331182)
